# Nuclear PLD1 combined with NPM1 induces gemcitabine resistance through tumorigenic IL7R in pancreatic adenocarcinoma

**DOI:** 10.20892/j.issn.2095-3941.2023.0039

**Published:** 2023-06-27

**Authors:** Danqi Fu, Jingrui Yan, Zhaoyu Zhang, Yang Liu, Xiaoqing Ma, Jinsheng Ding, Shengyu Yang, Ran Zhao, Antao Chang, Chuntao Gao, Jing Liu, Tiansuo Zhao, Xiuchao Wang, Chongbiao Huang, Song Gao, Ying Ma, Bo Tang, Yukuan Feng, Hongwei Wang, Jihui Hao

**Affiliations:** 1 Department of Pancreatic Cancer, Tianjin Medical University Cancer Institute and Hospital, National Clinical Research Centre for Cancer, Key Laboratory of Cancer Prevention and Therapy, Tianjin 300060, China; 2 Department of General Surgery, Changzheng Hospital, Naval Medical University (Second Military Medical University), Shanghai 200003, China; 3 Department of Cellular and Molecular Physiology, Penn State College of Medicine, Hershey, Pennsylvania 17033, USA

**Keywords:** Gemcitabine resistance, pancreatic ductal adenocarcinoma, phospholipase D1, nucleophosmin 1, CRISPRa library

## Abstract

**Objective:**

Pancreatic ductal adenocarcinoma (PDAC) is a highly malignant gastrointestinal cancer with a 5-year survival rate of only 9%. Of PDAC patients, 15%-20% are eligible for radical surgery. Gemcitabine is an important chemotherapeutic agent for patients with PDAC; however, the efficacy of gemcitabine is limited due to resistance. Therefore, reducing gemcitabine resistance is essential for improving survival of patients with PDAC. Identifying the key target that determines gemcitabine resistance in PDAC and reversing gemcitabine resistance using target inhibitors in combination with gemcitabine are crucial steps in the quest to improve survival prognosis in patients with PDAC.

**Methods:**

We constructed a human genome-wide CRISPRa/dCas 9 overexpression library in PDAC cell lines to screen key targets of drug resistance based on sgRNA abundance and enrichment. Then, co-IP, ChIP, ChIP-seq, transcriptome sequencing, and qPCR were used to determine the specific mechanism by which phospholipase D1 (PLD1) confers resistance to gemcitabine.

**Results:**

PLD1 combines with nucleophosmin 1 (NPM1) and triggers NPM1 nuclear translocation, where NPM1 acts as a transcription factor to upregulate interleukin 7 receptor (IL7R) expression. Upon interleukin 7 (IL-7) binding, IL7R activates the JAK1/STAT5 signaling pathway to increase the expression of the anti-apoptotic protein, BCL-2, and induce gemcitabine resistance. The PLD1 inhibitor, Vu0155069, targets PLD1 to induce apoptosis in gemcitabine-resistant PDAC cells.

**Conclusions:**

PLD1 is an enzyme that has a critical role in PDAC-associated gemcitabine resistance through a non-enzymatic interaction with NPM1, further promoting the downstream JAK1/STAT5/Bcl-2 pathway. Inhibiting any of the participants of this pathway can increase gemcitabine sensitivity.

## Introduction

Pancreatic ductal adenocarcinoma (PDAC) is a highly malignant gastrointestinal cancer with a 5-year survival rate of only 9%^1,2^. Of patients with PDAC 15%–20% are eligible for radical surgery because most patients are at a locally advanced or metastatic stage at the time of diagnosis^3–5^. Gemcitabine is an important chemotherapeutic agent for PDAC treatment; however, the efficacy of gemcitabine is limited due to the high incidence of drug resistance in PDAC^6–8^. The disease control rate (DCR) with gemcitabine-based chemotherapy is 65.8%, and resistance to gemcitabine treatment is a significant cause of poor prognosis^3^. Accordingly, reducing gemcitabine resistance is essential to improving the survival of patients with PDAC.

Gemcitabine [2′,2′-difluoro 2′-deoxycytidine (dFdC)] is a deoxycytidine analog, the cytotoxic activity of which is achieved by inhibiting DNA synthesis and preventing DNA repair^9^. Equilibrative nucleoside transporter 1 (ENT1), concentrative nucleoside transporter (CNT) 1, and CNT3 are gemcitabine transporters, the upregulation of which enhances the cytotoxic activity of gemcitabine *in vitro* and *in vivo*. Gemcitabine resistance is related to the metabolism of gemcitabine^9^. Indeed, the cytotoxic effects of gemcitabine are mainly exerted through the gemcitabine metabolites [dFdC diphosphate (dFdCDP) and dFdC triphosphate (dFdCTP)]. Moreover, numerous proteins regulate the transport and formation of the two metabolites, and we have reported that several oncogenes also interfere with gemcitabine-induced apoptosis^10^. To screen potential gemcitabine-resistance genes, we applied clustered regularly interspaced short palindromic repeats (CRISPR) to activate gene transcription. Many technologies are available for pooled screening, such as RNAi, CRISPR knock-out (ko), CRISPR interference (i), and CRISPR activation (a)^11^. Our study used CRISPRa to activate gene expression *via* dCas9 fusion with transcriptional activators. CRISPRa achieves robust increases in gene expression by recruiting VP64 and multiple other activators to the dCAS9 VPR system under the guidance of sgRNA^12^. Because CRISPRa can only perform its functions at a transcriptional start site, the frequency of off-target effects is lower than the RNAi method. Additionally, second-generation CRISPRa base editing systems combine Cas9 with mRNA editing enzymes to perform base editing more efficiently.

In this study we performed genome-wide CRISPRa/dCas9 overexpression screening in PDAC cells with gemcitabine and vehicle control treatments to systematically evaluate the essential mechanisms of gemcitabine resistance. We identified phospholipase D1 (PLD1), a transphosphatidylase, that primarily functions to hydrolyze lipid [phosphatidylcholine (PC)] to generate the dynamic lipid second messenger [phosphatidic acid (PA)] and choline dehydrocholate under physiologic conditions^13^. The product of PLD1, PA, is a dynamic lipid secondary messenger involved in a broad spectrum of cellular functions, including but not limited to metabolism, migration, and exocytosis^14^.

PLD1 may serve as a potential therapeutic target due to PLD1-promoted tumor proliferation and migration^15–20^. Few studies have investigated the relationship between PLD1 and chemotherapy in cancer^21,22^. To the best of our knowledge, the role of PLD1 in developing chemotherapy-resistant PDAC has yet to be explored. Therefore, we aimed to examine the role of PLD1 in chemotherapy-resistant PDAC in the current study.

## Materials and methods

### Cell culture

Human PDAC cell lines (BxPC-3, PANC-1, and SW-1990) and the human embryonic kidney cell line, HEK 293, were obtained from the Type Culture Collection Committee of the Chinese Academy of Sciences (Shanghai, China). The Human PDAC cell line, MIA PaCa-2, was obtained from the American Type Culture Collection (ATCC; Manassas, VA, USA). *Mycoplasma* contamination was excluded in all cell lines. The cells were cultured in Dulbecco’s Modified Eagle Medium (DMEM; C11995500BT, Gibco, Grand Island, CA, USA) or RPMI-1640 (C11875500BT; Gibco, Grand Island, CA, USA) basic medium with 10% fetal bovine serum (FBS; 10099141C, Gibco, Grand Island, CA, USA) at 37°C in a humidified at 5% CO_2_.

### Patients and tissue samples

We collected 100 PDAC and adjacent pancreas tissues from patients who underwent radical surgery following a histologic diagnosis of PDAC in 2010 at Tianjin Medical University Cancer Institute and Hospital (Tianjin, China). The data from these patients were retrospectively collected and included age, gender, tumor size, regional lymph node status, TNM stage, pathologic type, and differentiation. Among the 100 patients, all necessary details were available for 97.

### Ethics statement

The Ethics Committee at the Tianjin Medical University Cancer Institute and Hospital approved the use of the specimens and patient information (approval no. Ek2021173). All patients provided informed written consent for the use of their specimens and disease information for future investigations according to the Ethics Committee and in accordance with the recognized ethical guidelines of the Declaration of Helsinki.

### Immunochemistry (IHC)

IHC analysis of the tumor tissues from the patients with PDAC or the mouse models for PLD1 (#sc-28314; Santa Cruz Biotechnology, Inc., Santa Cruz, CA, USA), interleukin 7 receptor [(IL7R), #ab95024; Abcam, Cambridge, UK], and Ki-67 (#ab16667; Abcam) were performed using a DAB substrate kit (#ZLI-9018; ZSGB-BOI, Beijing, China). The score was determined by multiplying the staining intensity scores by the staining extent scores. The final score ranged from 0-12. The staining intensity score was as follows: 0, negative; 1, low; 2, medium; and 3, high. The staining extent score was scored as follows: 0, 0% stained; 1, 1%–25% stained; 2, 26%–50% stained; 3, 26%–75% stained; and 4, 76%–100% stained. Five random fields (100 × magnification) were evaluated under a light microscope. The IHC score was determined independently by two pathologists who were blinded to patient clinical features and outcomes.

### Immunofluorescence (IF)

Cells were fixed with ice-cold 4% paraformaldehyde fix solution (#P1110; Solarbio, Beijing, China) for 2 h and permeabilized with 0.1% Triton X-100 (#T8200; Solarbio, Beijing, China) for 1 h. Fixed cells were incubated for 1 h in 1% BSA, stained with primary antibodies overnight at 4 °C, and exposed to secondary antibodies for 2 h at 37 °C. The primary antibodies used in the IF assay were IL7R (#ab180521, 1:200; Abcam, Cambridge, UK), PLD1 (#sc-28314, 1:200; Santa Cruz Biotechnology, Inc., Santa Cruz, CA, USA), and nucleophosmin 1 [(NPM1), ab10530, 1:200; Abcam, Cambridge, UK]. Alexa Fluor 488 (#A12379, 1:1000; Invitrogen, Carlsbad, CA, USA) and Alexa Fluor 594 (#A12381, 1:1000; Invitrogen, Carlsbad, CA, USA) were used as secondary antibodies. Cell nuclei were stained with DAPI (#0100-20; SouthernBiotech, Birmingham, Alabama, USA) for 5 min. Ten representative non-overlapping confocal images were obtained with a Zeiss LSM 700 confocal microscope (Oberkochen, Baden-Württemberg, Germany) using the 20 × or 40 × objective.

### Real-time quantitative (PCR)

Total RNA was extracted from cells or tissues using TRIzol (#15596026; Invitrogen) and converted to cDNA using TaqMan reverse transcription reagents (TaqMan, Beijing, China). Next, qPCR was performed using SYBR Green Master mix (#b21203; Biomake, Beijing, China). The products of semi-quantitative PCR were detected by agarose gel electrophoresis with *GAPDH* as a loading control. The primers used are listed in **Table 1**.

**Table 1:** Primers for sequencing of CRISPRa library and PCR

Primer name	Primer sequence (5′–3′)
sgRNAactseqs1F	AATGGACTATCATATGCTTACCGTAACTTGAAAGTATTTCG
sgRNAactseqs1R	CTTTAGTTTGTATGTCTGTTGCTATTATGTCTACTATTCTTTCC
PLD1-F	CCCAGCGATCCCAAGATACAA
PLD1-R	GACAGCCGGAGAGATACGTCT
GSTT2-F	TGGCATCCCCTTAGAGCTG
GSTT2-R	CTTGAGCGTCGGCAGTTTC
NLRP1-F	GCAGTGCTAATGCCCTGGAT
NLRP1-R	GAGCTTGGTAGAGGAGTGAGG
IL7R-F	CCCTCGTGGAGGTAAAGTGC
IL7R-R	CCTTCCCGATAGACGACACTC

### Apoptosis assay

PDAC cell lines were analyzed using an Annexin-V FITC/PI double-staining method and a commercial kit (556570; BD Biosciences, NYC, NYC, USA) according to the manufacturer’s instructions. Cells were collected, washed with phosphate-buffered saline (PBS), suspended in 100 μL of binding buffer, and stained with 5 μL of phycoerythrin (PE)–Annexin-V and 5 μL of 7-AAD for 15 min in the dark. The stained cells were analyzed immediately. FACScan analyzed a minimum of 5000 cells with Cell Quest software (Becton-Dickinson, NYC, NYC, USA) for acquisition and analysis.

### Cell colony formation assays

The PDAC cells were diluted and replaced in 6-well plates (1000 cells per well). After incubation for 14 d, cell clones that had formed from individual cells were observed with an unaided eye, fixed with 4% paraformaldehyde for 15 min, then stained with crystal violet solution (#V5262; Sigma, St. Louis, MO, USA) for 30 min. The colony-forming efficiency (%) was calculated by the colony number-to-cell seeding number ratio.

### Co-immunoprecipitation (Co-IP)

Co-IP was performed using protein A/G magnetic beads (#B23202; Bimake, Houston, Texas, USA) according to the manufacturer’s instructions. The cell nucleus or cytoplasm protein extracts were prepared using NE-PER™ Nuclear and Cytoplasmic Extraction Reagents (#78835; Thermo Fisher, Waltham, MA, USA). PLD1 (#3832s; Cell Signaling Technology, Boston, MA, USA) and NPM1 antibody (#10306-1-AP; Proteintech, Chicago, Illinois, USA) were separately conjugated with protein A/G magnetic beads in cell lysis buffer for 2 h at 20–30°C. The antibody-bead complexes were collected using the magnetic frame and incubated with cell extracts overnight at 4°C. The antibody-bead-protein complexes were collected using the magnetic frame and washed 5 times with excess PBS containing 0.1% NP-40. The final precipitate was dissolved in sodium dodecyl sulfate (SDS) sample buffer and analyzed by SDS-polyacrylamide gel electrophoresis (PAGE) and Western blot.

### Chromatin Immunoprecipitation (ChIP) and ChIP: reChIP assay

ChIP assays were performed using a ChIP kit (Millipore, Boston, MA, USA) according to the manufacturer’s instructions. Briefly, MIA PaCa-2 cells were immunoprecipitated with anti-NPM1 antibody. Cells were first immunoprecipitated with anti-NPM1 antibody, then immunoprecipitated again with anti-PLD1 antibody for the ChIP: reChIP analysis. The immunoprecipitated products were detected using an RT-PCR assay and agarose gel electrophoresis.

The Dual-Luciferase^®^ Reporter Assay System (#E1910; Promega, Madison, WI, USA) was used for luciferase analyses according to the manufacturer’s instructions. MIA PaCa-2 cells transfected with plv-PLD1 or control vector (plv-vector) were transfected with the following vectors: pGL3-PLD1-EBS (WT); pGL3-PLD1-EBS1-mutation; and pGL3-empty vector (pGL3.1EV). The cells were subjected to dual luciferase analysis 48 h later. The results are expressed as fold-change relative to the cells transfected with the control vector after normalization to Renilla luciferase activity.

### PLD activity assay

A PLD Assay Kit (#MAK137; Sigma) was used following the manufacturer’s recommended protocol. Specifically, PLD hydrolyses PC to choline, which is determined using choline oxidase resulting in a colorimetric (570 nm)/fluorometric (λ_ex_ = 530/λ_em_ = 585 nm) product proportional to the PLD activity in the sample. Three technical and biological replicates were used in the PLD activity assay. The initial and final absorbances were measured at 570 nm.

### Cell Counting Kit-8 (CCK-8) Assay

PDAC cells were plated (1000 cells/well) in 96-well plates for the CCK-8 assay. After culturing for 12 h, the cells were treated with a gradient concentration of drugs. Then, the cells were incubated at 37 °C in an incubator with 5% CO_2_ for 48 h. Finally, cell viability was determined using the CCK-8 assay (#B34302; Biomake). The cytotoxic effect of the drugs on the PDAC cells was measured using an immunosorbent instrument at 450 nm (BioTek Synergy H1; Winooski, Vermont, USA).

### Gene set enrichment analysis (GSEA)

GSEA was performed with Java GSEA software (v4.2.3; https://www.broadinstitute.org/gsea). Normalized gene expression profiles were ranked with signal-to-noise metrics, and enrichment scores were calculated with a random gene set permutation of 1000. Statistical significance was set at a nominal *P*-value (Nom *P*-value) < 0.05.

### Statistical analysis

Data were analyzed using PRISM v8.0 Software (GraphPad Prism8). Binary comparisons between two treatment arms were made using an unpaired, two-tailed Student *t*-test. Differences at the 95% confidence level (*P* < 0.05) were statistically significant. Analysis of statistically significant changes in Kaplan–Meier survival curves was made using a log-rank test. Differences at the 95% confidence level were considered statistically significant. For treatment studies, the primary endpoints were death due to tumor volume > 3000 mm^3^ and r ≥ 20% loss in mouse body weight. The sample size was estimated based on an expected effect size of 0.2 for tumor volume changes with a type I error rate of 0.05. For blocking studies, the sample size was prospectively estimated using an anticipated effect size of 0.5 between the tracer uptake in blocked versus unblocked tumors and a type I error rate of 0.05.

Details related to the other assays are described in the **Supplementary materials and methods** (**Supplementary Doc S1**).

## Results

### Whole genome functional screening for gemcitabine resistance genes

**Figure 1:**
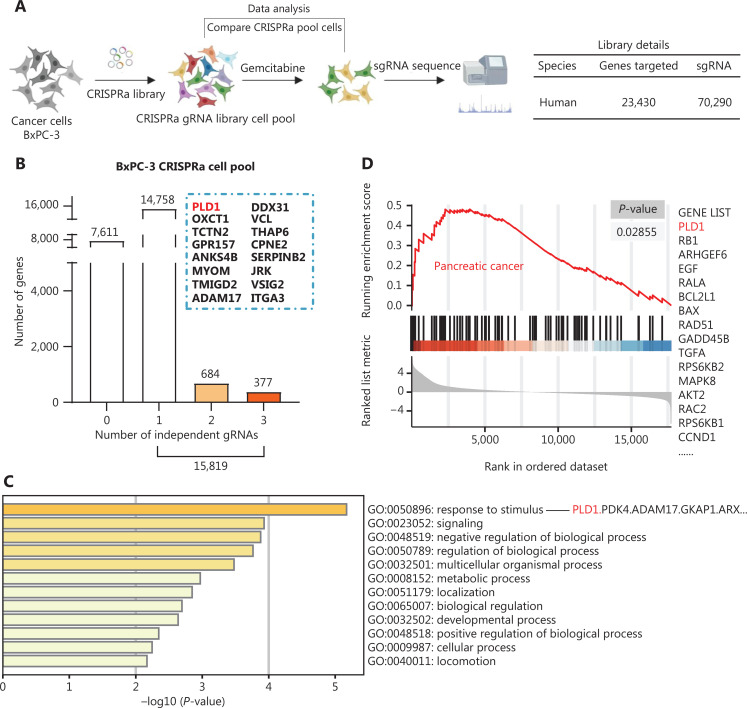

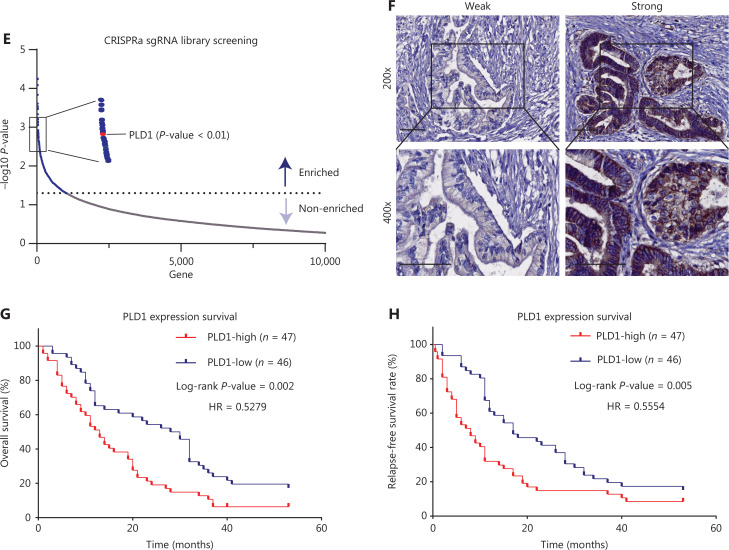
CRISPRa library screening identified PLD1 as a molecular driver for gemcitabine resistance. (A) Schematic diagram illustrating the workflow of genome-wide CRISPRa/dCas9 overexpression library screening (CRISPR; clustered regularly interspaced short palindromic repeats). A human genome-wide CRISPRa/dCas9 overexpression library containing 70,290 sgRNAs (23,430 genes) was packed into lentiviral particles and transduced into dCas9-overexpressing BxPC-3 cells at a low multiplicity of infection (MOI). The sgRNA-transduced BxPC-3 cells were selected by puromycin to generate a mutant cell pool. Mutant cells were cultured in vehicle and gemcitabine for 3 days to screen resistant genes. Genomic DNA extracted from the treated BxPC-3 cells and the sgRNA fragment was amplified by PCR. The sgRNA copy number was determined by high-throughput sequencing and analyzed using the MAGeCK v0.5.7 algorithm. (B) Target genes were classified as an independent gRNA number during gemcitabine treatment. The group that enriched three independent gRNAs contained PLD1. (C) Gene Ontology revealed that PLD1-targeted genes were enriched in the pathway of tumor cell responses to stimuli. (D) Gene Set Enrichment Analysis (GSEA) showed that genes involved in pancreatic cancer signaling were significantly upregulated in gemcitabine-resistant cells (day 3) when compared to the parental cells (day 0), and PLD1 could be found in the signaling pathway. (E) A scatter diagram revealed that PLD1-targeting sgRNAs were positively selected for during gemcitabine treatment, suggesting that PLD1 is an indispensable gene for PDAC cells to survive gemcitabine treatment. (F). Representative IHC images of PLD1 expression using human PDAC tissue sections (*n* = 93), bar = 200 μm. (G, H) Kaplan–Meier OS and RFS for different levels of PLD1 based on the log-rank statistic test (*P* < 0.001).

To identify genes involved in developing gemcitabine resistance in PDAC, we utilized a genome-wide CRISPRa/dCas9 transcriptional activation library that included 70,290 sgRNAs targeting 23,430 genes in 3 independent replicates (**Supplementary Figure S1A**). The BxPC-3 PDAC cell line was selected to construct a CRISPRa library cell line due to the sensitivity to gemcitabine (**Figure 1A**). We treated CRISPRa library pool cells with saline or gemcitabine for 3 d and used next-generation sequencing to identify enriched screen gemcitabine-resistant genes and sgRNA sequences. After the screening, a subset of sgRNAs targeting 15,819 genes was observed to be prominent (*P* < 0.05) in the gemcitabine-treated cells compared to the vehicle control cells, indicating that these genes may be potential drivers of gemcitabine resistance (**Figure 1B**). Gene Ontology (GO) analysis indicated that these genes were enriched in cell responses to stimuli, cell signaling, and cell metabolic processes (**Figure 1C**). In addition, GSEA revealed that the PDAC pathway was significantly activated in the gemcitabine-resistant samples (**Figure 1D**).

PLD1 was listed in the two gene enrichment sets (GO and GSEA) because PLD1-targeting sgRNAs underwent the most positive selection during gemcitabine treatment, suggesting that PLD1 is an indispensable gene for the survival of PDAC cells in response to gemcitabine treatment (**Figure 1E**). PLD1 also enriched three independent gRNAs by classifying the target genes as an independent gRNA number during gemcitabine treatment (**Figure 1B**). Moreover, we analyzed the level of PLD1 mRNA expression in gemcitabine-sensitive and -resistant cell lines using RNA-seq. We continuously added 10 or 100 nM of gemcitabine to MIA PaCa-2 cells. The surviving cells were amplified and propagated for the gemcitabine-sensitive (GS) and gemcitabine-resistant (GR) cell lines. A significant difference was detected between the levels of PLD1 mRNA in GS and GR cell lines; specifically, the GR cell line had more than twice the PLD1 mRNA compared to the GS cell line (**Supplementary Figure S1B**). These observations indicated that PLD1 may have a critical role in developing gemcitabine resistance.

### PLD1 was identified as a key target that induces gemcitabine resistance in PDAC cell lines

PLD1 was expressed at higher levels in PDAC tissues than para-cancer tissues at the protein and RNA levels (**Supplementary Figure S1C–S1E**). As the degree of tissue malignancy increased, the level of PLD1 expression increased accordingly (**Supplementary Figure S1F**). By analyzing PLD1 expression levels in the tissue microarrays (TMAs) of 93 patients with PDAC who received chemotherapeutic regimens, including gemcitabine, we showed that the expression of PLD1 was inversely correlated with overall survival (OS) and relapse-free survival (RFS; **Figure 1F–1H**). Analyses of the public database and immunohistochemical staining of the TMAs suggested an inverse correlation between PLD1 expression and prognosis. This evidence suggests that PLD1 was a significant biomarker for predicting the efficacy of gemcitabine chemotherapy.

**Figure 2:**
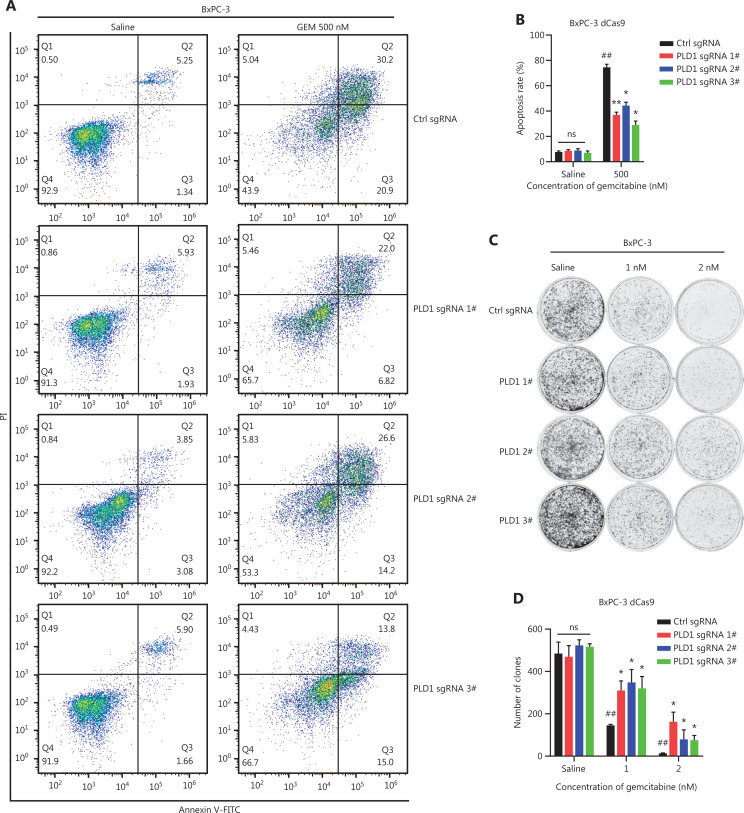

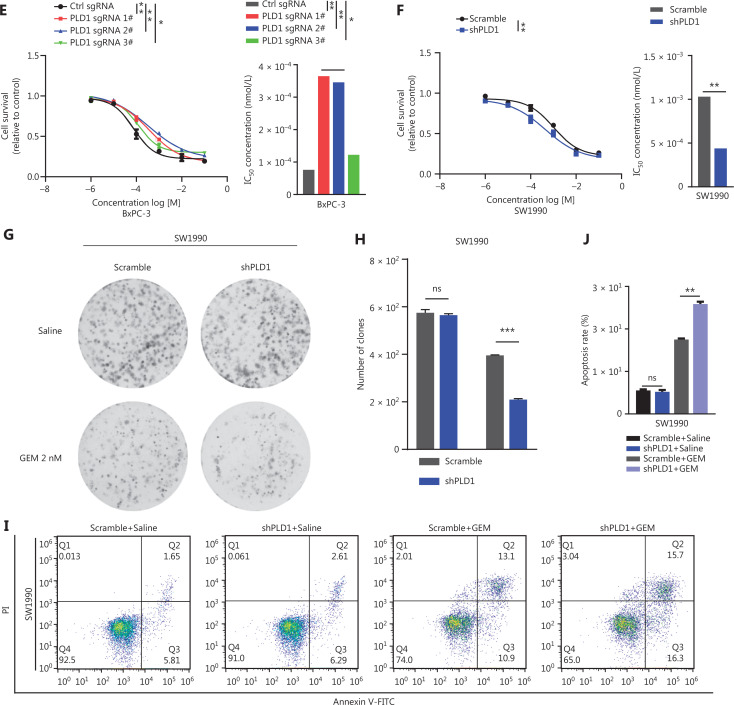

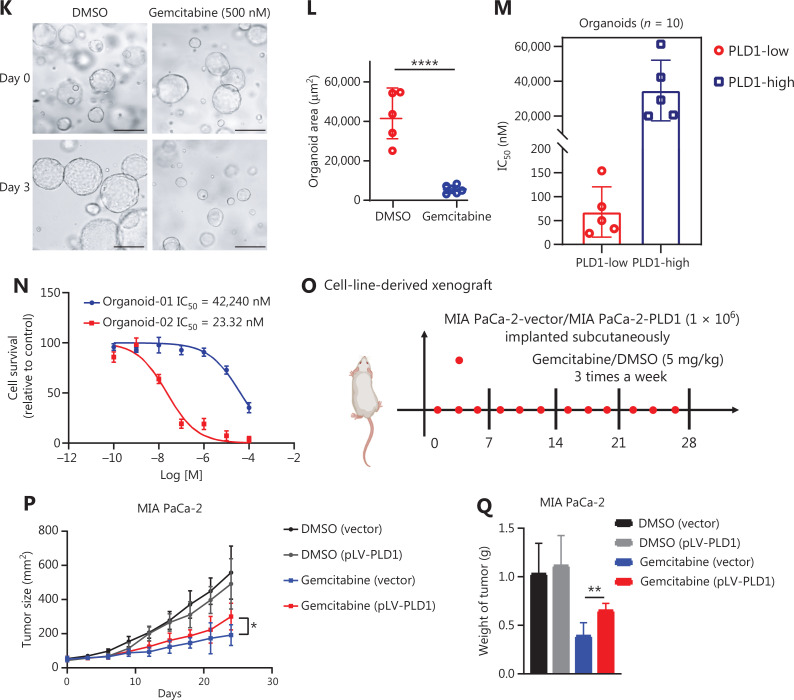
PLD1 promotes gemcitabine resistance in PDAC. (A, B, I, J) Flow cytometry was performed to detect the apoptosis rates of cell lines treated with 500 nM gemcitabine or saline for 72 h. The corresponding statistics are presented in the histogram. (C, D) Representative images and quantification of colony formation in cell lines that were treated for 72 h with gemcitabine or saline revealed that overexpression of PLD1 increased gemcitabine resistance. (E, F) Cell viability was examined using the CCK-8 assay after a 72-h treatment with gemcitabine (*n* = 3, per group). PLD1 significantly increased gemcitabine resistance in PDAC cells. (G, H) Cell cloning capability was analyzed using the colony formation assay in PLD1-overexpressing stable cell lines and PLD1 knockdown cell lines. (K-N) Cell viability was detected by Cell titer glo (CTG) after PDAC organoids were treated with gemcitabine for 72 h. Organoid area statistics are presented in the scatterplot. Organoids derived from patients were classified into two groups by PLD1 expression based on IHC results. The high-PLD1 expression group showed stronger gemcitabine resistance, bar = 200 μm. (O, P) The indicated tumor cells were subcutaneously transplanted into the nude mouse model to develop tumors (*n* = 6 per group). After 7 days, the mice were treated with saline or gemcitabine using a caliper to measure tumor volumes every 3 days. Finally, the mice were sacrificed, and tumors were harvested to provide representative images of the tumors. (Q) The statistics of tumor weight in mice (*n* = 6, per group). *P < 0.05; **P < 0.01; ***P < 0.001; ns, not significant.

To validate the induction of gemcitabine resistance by PLD1 and the reliability of our CRISPRa library screening *in vitro*, we constructed a PLD1 stable overexpression (OE) cell line by infecting BxPC-3-dCas9 cells with three sgRNAs that had the same library sequence. Western blot confirmed that all sgRNA sequences effectively induced OE of PLD1 in BxPC-3 cells (**Supplementary Figure S2A, S2B**) and PLD1 sgRNA OE cell lines exhibited apparent gemcitabine resistance effects. First, flow cytometry detected cell apoptosis under saline- and gemcitabine-treated conditions. The apoptosis rate of PLD1 OE cell lines was nearly the same as control cells in the non-gemcitabine medium, whereas the PLD1 OE cell lines demonstrated apoptosis inhibition under gemcitabine treatment (**Figure 2A, 2B**). The results of colony formation assays also showed gemcitabine resistance when PLD1 was overexpressed (**Figure 2C, 2D**). Finally, the IC_50_ was monitored using a CCK-8 assay. Stable transfectants with high levels of PLD1 showed increased viability after gemcitabine treatment (**Figure 2E, Supplementary Figure S2C**).

Additionally, we constructed sequence-specific shRNAs to silence the expression of PLD1 in the PANC-1 and SW1990 cell lines, and transfected pLV-PLD1 lentivirus into BxPC-3 and MIA PaCa-2 cell lines to construct cell lines with stable expression of PLD1. Cell lines were selected based on the level of PLD1 expression. The levels of PLD1 mRNA and protein expression were validated in all cell lines in which PLD1 had been overexpressed or knocked down. BxPC-3-dCas9 and PLD1 OE cell lines consistently presented similar results, with the knockdown of PLD1 showing opposite effects (**Figure 2F–2J, Supplementary Figure S2D–S2H**).

Next, we sought to confirm this finding using a human PDAC organoid model. We collected 10 PDAC tissues from patients who had received gemcitabine treatment to construct human PDAC organoids. Based on the Western blot results from these organoid models, we classified the models into two groups based on the level of PLD1 expression (**Figure 2K–2M**). The Cell Titer Glo Luminescent Cell Viability Assay (promega, Madison, WI, USA) was used to calculate the IC_50_ (**Figure 2N**). Our results strongly indicated that organoids with lower PLD1 expression had lower viability under gemcitabine treatment. Nude mice were xenografted with MIA PaCa-2 pLV-vector and MIA PaCa-2 pLV-PLD1 cell lines. Mice were treated with gemcitabine or saline when tumors became palpable on day 7. We measured tumor volumes during treatment until the tumor volumes reached 500 mm^3^, then calculated tumor rejection ratios to rule out proliferation interference caused by PLD1 (**Figure 2O, 2P**). In addition, the weights of the four tumor groups were measured for comparison. The MIA PaCa-2 pLV-PLD1 tumor treated with gemcitabine was clearly heavier than the MIA PaCa-2 vector tumor treated with gemcitabine (**Figure 2Q**). Overall, our results indicate that PLD1 significantly increased gemcitabine resistance of PDAC cells *in vitro* and *in vivo*.

### PLD1 induced gemcitabine resistance mainly through expression in the nucleus of PDAC cells

PLD is a transphosphatidylase that primarily works to hydrolyze PC to generate PA and choline dehydrocholate^12^. In previous studies, PLD1 has been shown to perform multiple functions *via* PA^21^. We selected an agonist and an inhibitor of PLD1 to determine whether PLD1 enhances gemcitabine resistance. Based on previous studies, we selected Vu0155069 as a selective inhibitor of PLD1 and phorbol-12-myristate-13-acetate (PMA) as an agonist^22–24^.

**Figure 3:**
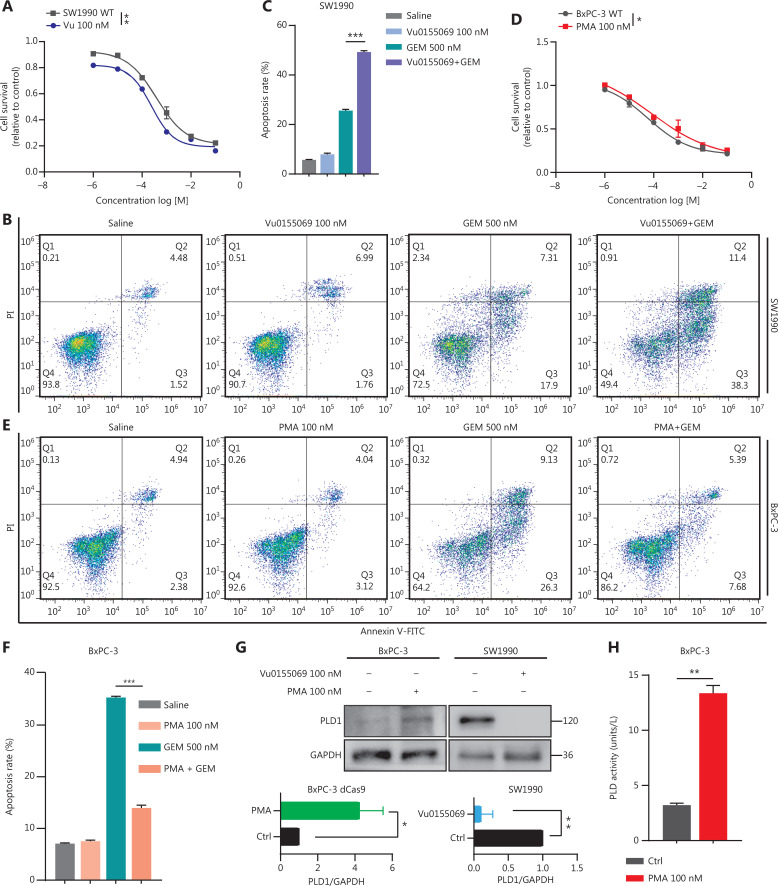

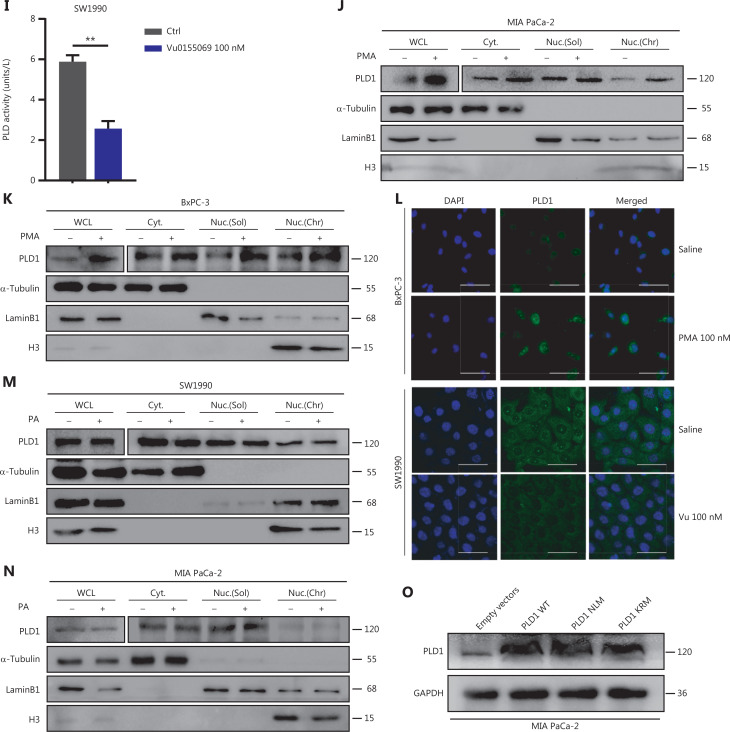

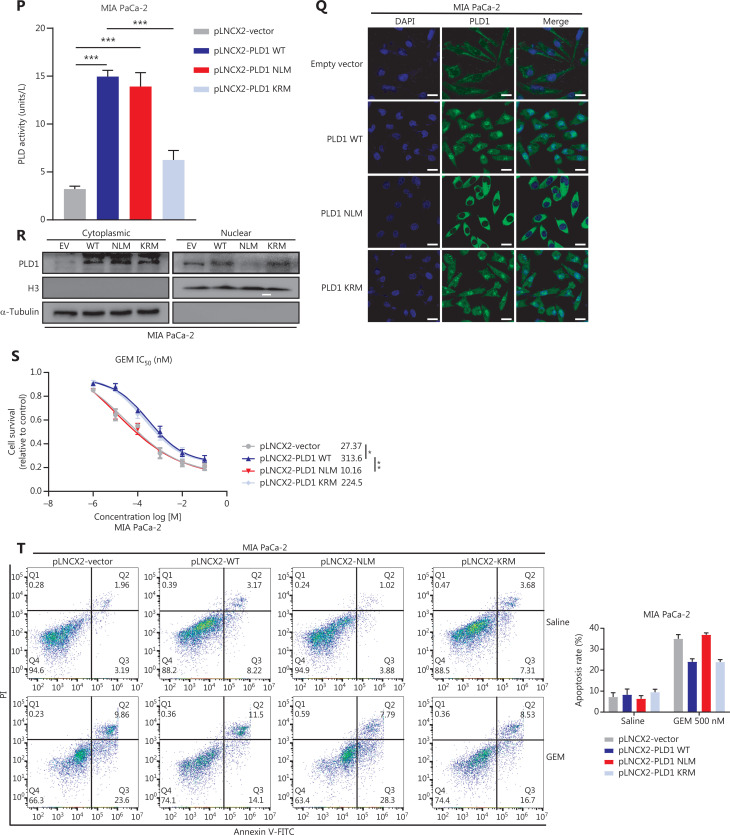
Intranuclear PLD1 plays a critical role in gemcitabine resistance. (A) Cell viability was tested using the CCK-8 assay after a 72-h treatment with gemcitabine (*n* = 3 per group). Changing the activity of PLD1 by pre-treatment with a PLD1 inhibitor (Vu0155069) significantly altered gemcitabine resistance in PDAC. (B, C) Flow cytometry was performed to detect the apoptosis rates of the cell lines pre-treated with Vu0155069 and treated with 500 nM gemcitabine or saline for 72 h. The corresponding statistics are presented in the histogram. (D) Cell viability was tested by CCK-8 assay after 72 h treatment with gemcitabine (*n* = 3 per group). Changing the activity of PLD1 by pre-treatment with a PLD1 agonist (PMA) significantly altered gemcitabine resistance in PDAC. (E, F) Flow cytometry was performed to detect the apoptosis rates of the cell lines pre-treated with Vu0155069 and treated with 500 nM gemcitabine or saline for 72 h. The corresponding statistics are presented in the histogram. (G-I) Both the PLD1 agonist and inhibitor can change both the protein level and enzyme activity level of PLD1. (J, K) Nucleocytoplasmic separation Western blot was performed to measure intranuclear and extranuclear PLD1 distribution after pre-treatment with PMA. (L) Representative immunofluorescence, bar = 50 μm (IF) images are shown with staining in antibodies against PLD1 (green) in the cell lines shown. Nuclei were stained with DAPI (blue). Nuclear PLD1 expression was increased after pre-treatment with the PLD1 agonist, PMA, and intranuclear PLD1 expression was decreased after pre-treatment with the PLD1 inhibitor-Vu0155069. (M, N) Nucleocytoplasmic separation Western blot was performed to measure intranuclear and extranuclear PLD1 distribution after pre-treatment with PMA. (O) The level of PLD1 protein expression in NLM and KRM cell lines as determined by Western blot. (P) Detection of the enzyme activity level in PLD1 NLM and KRM cell lines by PLD1 enzyme activity assay kit. (Q) Confocal microscopy showed the subcellular localization of PLD1 in PLD1 NLM and KRM cell lines, bar = 20 μm. (R) Nucleocytoplasmic separation Western blot was performed to measure intranuclear and extranuclear PLD1 distribution in PLD1 NLM and KRM cell lines. (S) Cell viability was examined using the CCK-8 assay after the treatment of GEM (*n* = 3, per group) in PLD1 nuclear localization signal mutation (NLM) and KRM cell lines. The PLD1 NLM cell line had a lower cell viability rate compared to the KRM cell line. (T) Flow cytometry was performed to detect the apoptosis rates of the indicated cell lines treated with 500 nM gemcitabine or saline for 72 h. The corresponding statistics are presented in the histogram. The PLD1 NLM cell line had significantly reduced gemcitabine resistance compared to the KRM cell line. *P < 0.05; **P < 0.01; ***P < 0.001.

First, to investigate the fully functional work of Vu0155069 and PMA, we displayed PLD1 protein expression with the two drugs pre-treatment in a time or concentration gradient by Western blot (**Supplementary Figure S3A–S3H**). Based on the results, 100 nM PMA or Vu0155069 was used to pre-treat cell lines for 24 h. Vu0155069 was used to pre-treat SW1990 cell lines to observe changes in gemcitabine resistance. The cytotoxic potential of gemcitabine (500 nM) was determined using a CCK-8 assay (**Figure 3A**). In contrast to the control group, Vu0155069 treatment significantly reversed the capability of PDAC to tolerate gemcitabine cytotoxicity. Moreover, flow cytometry apoptosis assays suggested that Vu0155069 pre-treatment increased the apoptosis rate of PDAC cells induced by gemcitabine (500 nM; **Figure 3B, 3C**). Treating BxPC-3 cells with PMA resulted in better cell viability than the control group (**Figure 3D**). Pre-treatment with PMA dramatically reduced apoptosis in the presence of gemcitabine (**Figure 3E, 3F**).

PLD1 hydrolyses PC to produce PA and choline dehydrocholate. To determine whether the classical enzyme activity pathway of PLD1 was responsible for the effect in promoting gemcitabine resistance, we treated MIA PaCa-2 and BxPC-3 cell lines with PA choline dehydrocholate, or a combination of both. The cell lines were pre-treated with 100 nM PA to test MAPKp44/42 pathway activation by Western blot. PA was absorbed into the cells and activated the MAPKp44/42 pathway (**Supplementary Figure S3I**). Interestingly, these treatments did not promote gemcitabine resistance in MIA PaCa-2 or BxPC-3 cells. Neither drug nor the combination of drugs function to elevate gemcitabine resistance (**Supplementary Figure S3J–3L**). To determine whether a novel gemcitabine resistance mechanism might exist, we confirmed that both Vu0155069 and PMA affected protein expression and enzyme activity levels of PLD1 by Western blot analysis and a PLD1 enzyme activity test kit (**Figure 3G–3I**)^23,24^. Our data indicated that gemcitabine resistance induced by PLD1 was achieved *via* a non-enzymatic pathway.

The classical enzyme activity pathway of PLD1 was not responsible for gemcitabine resistance; however, PMA and Vu0155069 significantly changed the cell viability ratio of the cells to which they were applied. Recent studies have reported the existence of a nuclear lipid metabolism gene related to cellular proliferation and migration^25–28^. To determine whether PLD1 is involved in nuclear regulation in PDAC, we first performed nucleocytoplasmic separation Western blot to examine the cytoplasmic and nuclear distribution of PLD1 after treatment with PA, choline, Vu0155069, and PMA. Interestingly, we found that PMA and Vu0155069 did affect the nuclear localization of PLD1 (**Figure 3J, 3K** and **Supplementary Figure S4A, S4B**) and the change in protein fractions as shown by immunofluorescence after pre-treatment of PMA and Vu0155069 (**Figure 3L**). The immunofluorescence results also confirmed this finding. Nevertheless, PLD1 expression changed upon PA and choline treatment, and nucleocytoplasmic separation Western blot supported this finding (**Figure 3M, 3N** and **Supplementary Figure S4C, S4D**). Because Vu0155069 and PMA had the potential to affect gemcitabine resistance, we hypothesized that the intranuclear activity of PLD1 might mediate gemcitabine resistance. To test our hypothesis, we constructed a nuclear localization sequence (NLS) mutation (NLM) PLD1 cell line that abolished PLD1 nuclear importation and mutations in enzyme viable sites (KRM) that cause the loss of PLD1 enzyme activity. The NLM-PLD1 cell line harbored five lysine-to-alanine substitutions (K553A, R555A, K556A, K559A, and K564A; **Supplementary Figure S4E**)^27^. We verified the level of PLD1 protein expression in empty-vector (EV), PLD1-WT (WT), NLM, and KRM cell lines by Western blot (**Figure 3O**). Moreover, IF analyzed by confocal scanning laser microscopy and PLD1 enzyme activity detection and nucleocytoplasmic separation Western blot revealed that the nuclear expression of PLD1 in the NLM cell line was nearly zero. In addition, the lysine-to-arginine substitution in the catalytic region of PLD1 greatly decreased the catalytic ability (**Figure 3P–3R**). Of the four cell lines, only the NLM cell line lost gemcitabine resistance. This finding strongly suggests that intranuclear PLD1 has a critical role in gemcitabine resistance (**Figure 3S, 3T**). In addition, the MIA PaCa-2 cell line was pre-treated with 50 nM gemcitabine for IF to detect the influence of gemcitabine. Gemcitabine increased PLD1 expression in the whole cell. When the total expression of PLD1 was significantly increased, more PLD1 translocated into the nucleus, which might increase PLD1-induced gemcitabine resistance (**Supplementary Figure S3M**).

### PLD1 bound NPM1 and shuttled PLD1 to the nucleus to induce gemcitabine resistance

**Figure 4:**
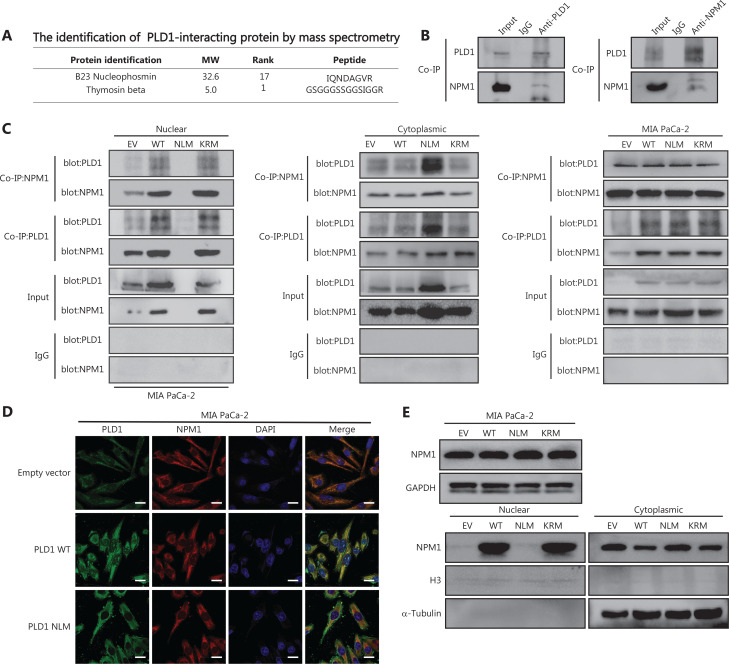

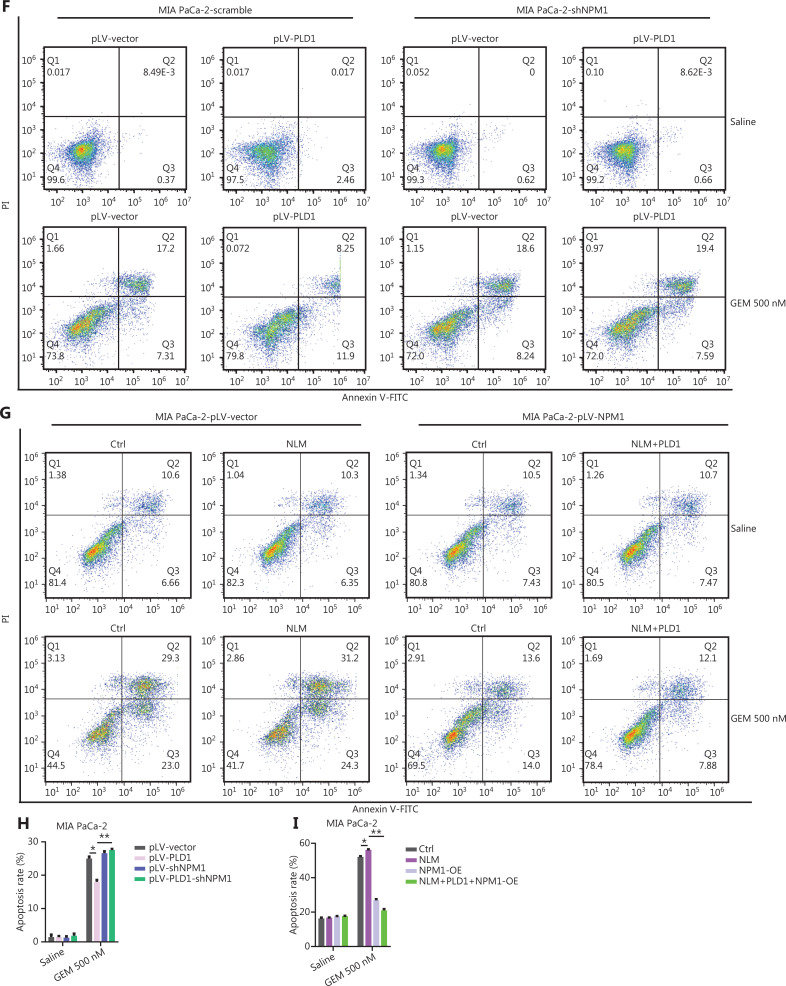
PLD1 bound NPM1 and shuttled NPM1 to the nucleus. (A) The results of immunoprecipitation-mass spectrometry (IP-MS) analysis. (B, C) Identification of PLD1-interacting proteins by coimmunoprecipitation and mass spectrometry. The different proteins precipitated from PLD1 and NPM1 antibodies in PLD1 overexpression cell lines were subjected to mass spectrometry analysis. The intracellular, cytoplasmic, and total cell interaction between coimmunoprecipitation assays in the indicated cell lines. (D) Representative immunofluorescence (IF) images showed staining with antibodies against PLD1 (green) and NPM1 (red) in the indicated cell lines. The nucleus was stained with DAPI (blue). PLD1 colocalizes with NPM1 in the nucleus; however, when PLD1 importation into the nucleus decreased, NPM1 importation was barely visible, bar = 200 μm. (E) Detection of nuclear and cytoplasmic NPM1 levels in PLD1, NLM, and KRM cell lines by Western blot. (F, G) Representative dot plot and statistical analysis of the frequency of CTRL (left), PLD1-OE (middle 1), NPM1-KD (middle 2), and PLD1-OE+NPM1-KD (right). (H, I) Representative dot plots and statistical analysis of the frequency of CTRL (left), NPM1-OE (middle 1), PLD1-KD (middle 2), and PLD1-KD+NPM1-OE (right). *P < 0.05; **P < 0.01.

As a result of our finding that intranuclear PLD1 is at the core of gemcitabine resistance, we conducted intranuclear immunoprecipitation-mass spectrometry (IPMS) analysis to identify nuclear proteins that interact with PLD1. Our mass spectrometry results revealed 110 binding proteins that bound to PLD1. Among these proteins, we focused on a molecule (NPM1) closely related to PLD1 intranuclear function in gemcitabine resistance. As a shuttling protein, NPM1 frequently shuttles between the cytoplasm and the nucleus. NPM1 ranked 17^th^ based on mass spectrometry results (**Supplementary Data File 1**, **Figure 4A**). We selected the nucleoplasmic shuttle protein, NPM1, for further testing and confirmed that PLD1 interacted with NPM1 *via* Co-IP experiments (**Figure 4B**). To further validate the relationship between PLD1 and NPM1, we performed Co-IP experiments in EV, WT, NLM, and KRM cell lines after nucleocytoplasmic separation. While PLD1 was not expressed in the nucleus in the NLM stable line, NPM1 expression in the nucleus was significantly reduced in the nucleocytoplasmic separation Western blot (**Figure 4C**).

Nuclear NPM1 levels increased along with PLD1, indicating that nuclear NPM1 levels co-localized in the nucleus. In addition, PLD1 and NPM1 bound each other when in the nucleus or cytoplasm. Detection of IF using confocal microscopy validated the co-localization of PLD1 with NPM1 (**Figure 4D**). As assessed by IF using confocal microscopy and nuclear and cytoplasmic extraction Western blot, we showed that more NPM1 could be induced in the nucleus with higher intranuclear expression of PLD1. In contrast, intranuclear NPM1 decreased significantly when NLM-PLD1 was localized exclusively in the cytosol. Our data showed that PLD1 interferes with the subcellular distribution of NPM1; however, NPM1 does not increase PLD1 levels (**Figure 4E**). To further explore whether NPM1 is a crucial downstream molecule of PLD1, we constructed stable shRNA constructs targeting NPM1 to perform an NPM1 blocking assay. NPM1 knockdown reversed the gemcitabine resistance induced by PLD1 overexpression (**Figure 4F, 4G**). Additionally, as the levels of PLD1 expression changed the subcellular distribution of NPM1 in the nucleus, the apoptosis rate of PLD1 knockout and NPM1 overexpression control cell lines was nearly the same as the PLD1 knockout cell line under gemcitabine chemotherapy.

Gemcitabine resistance cannot completely recover when NPM1 alone is overexpressed. Therefore, we simultaneously transfected an NLM cell line with Plv-PLD1 and Plv-NPM1. When PLD1 coordinated the import of NPM1 into the nucleus, the apoptosis rate was reduced significantly (**Figure 4H, 4I**). These data demonstrate that NPM1 is an essential downstream molecule for PLD1-mediated gemcitabine resistance.

### NPM1 combined with PLD1 transactivates IL7R expression in PDAC cells

To identify the key domains of PLD1 responsible for the interaction with NPM1, a group of PLD1 deletion constructs was assembled (**Supplementary Figure S5A**). The Co-IP results suggested that the PH domain-truncated PLD1 mutants (PX and PLD) completely lost NPM1 binding ability and the PH domain of PLD1 was sufficient to interact with NPM1 (**Supplementary Figure S5B**). Similarly, we generated a set of NPM1 deletion constructs (**Supplementary Figure S5C**). The Co-IP results revealed that C-terminalΔ3 was sufficient to interact with PLD1. Thus, we speculated that PLD1 interacts with the NPM1 C-terminal *via* the PH domain (**Supplementary Figure S5D**).

**Figure 5:**
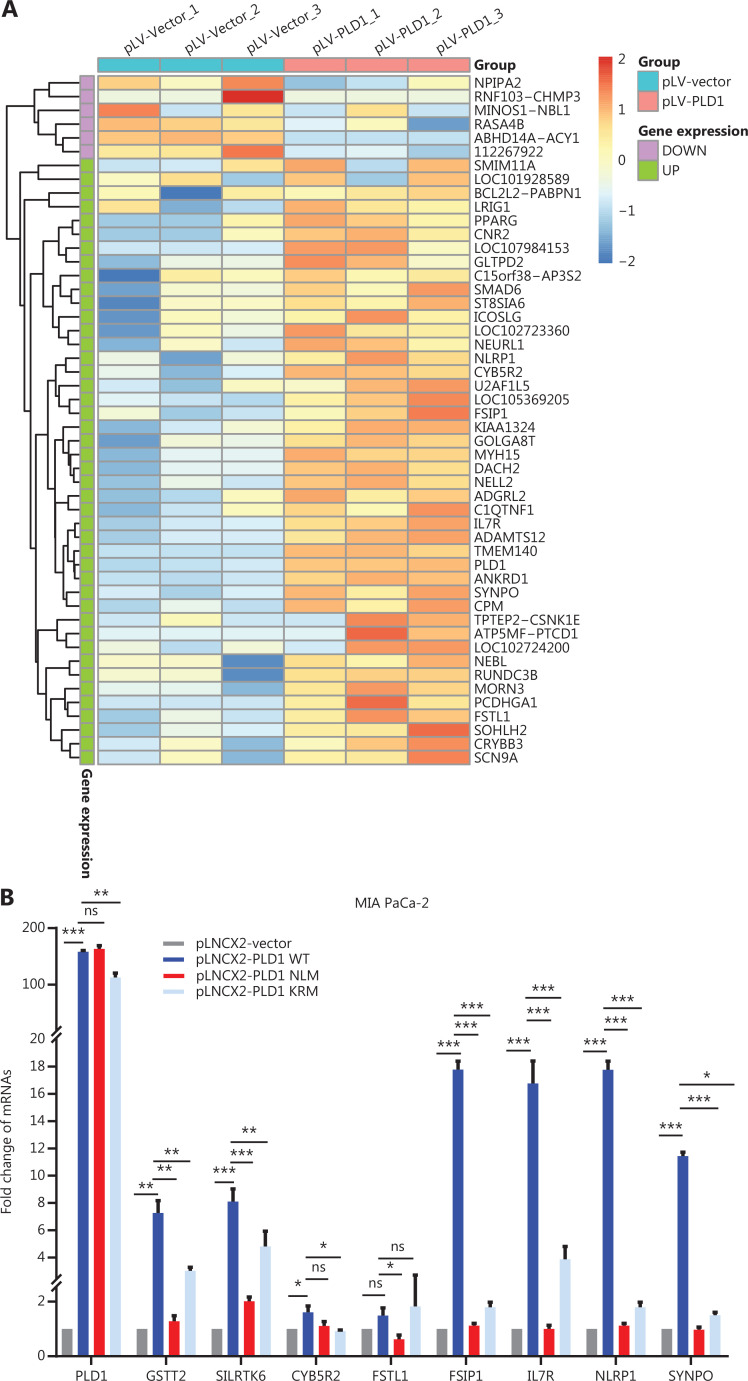

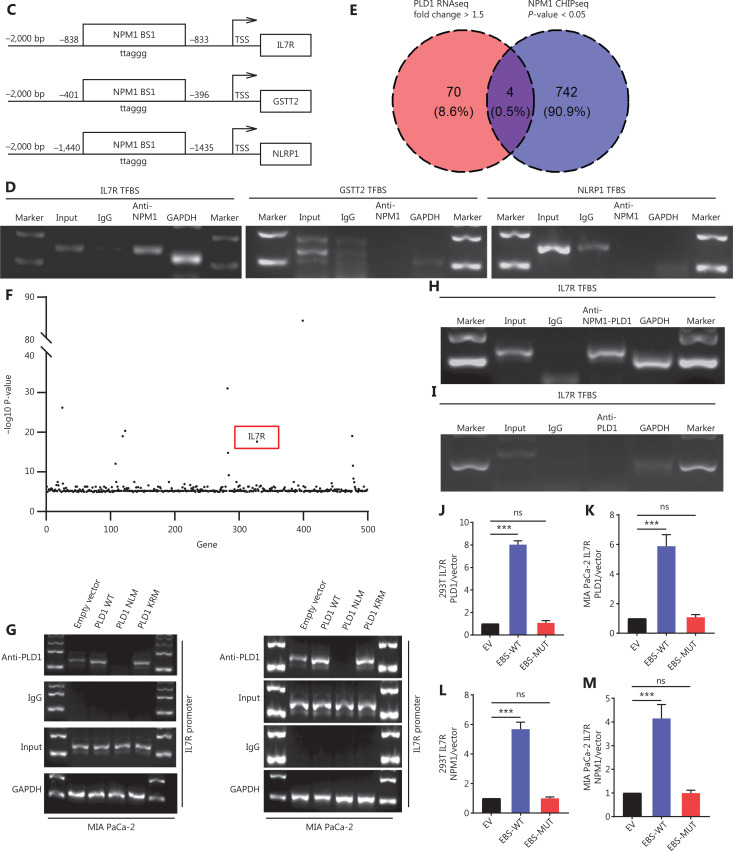
NPM1 and PLD1 bind at the promoter region of *IL7R.* (A) Heatmap showing the results of CTRL and PLD1-OE cell line RNA-seq. (B) Changes in gene expression profiling upon changes to PLD1 expression in the indicated cell lines. qPCR was performed to detect the transcriptional change in the genes selected from RNA-seq analysis. Relative expression is shown as a fold-change relative to GAPDH. (C, D) Binding of PLD1 to the promoters of IL7R, GSTT2, and NLRP1 in MIA PaCa-2-PLD1 determined by ChIP. Experiments were repeated three times independently. Representative data are shown. (E) Venn diagram in which PLD1 was identified as one of the core intersections of RNA-seq and CHIP-seq analyses. (F) Scatter plots indicated that NPM1 binds with the promoters of IL7R in CHIP-seq data. (G) Binding of NPM1 and PLD1 to the promoters of IL7R in empty vector, PLD1-WT, and PLD1-KRM by CHIP. (H) Binding of NPM1 and PLD1 to the promoters of IL7R in empty vector, PLD1-WT, and PLD1-KRM by RE-CHIP. (I) Binding of PLD1 to the promoter of IL7R was determined by chromatin immunoprecipitation in the NPM1 KD cell line. IgG was used as a negative control. Anti-GAPDH was used as a positive control. Representative results are shown. (J-M) The HEK 293T (left) and MIA PaCa-2 cells (right) were transfected with control or pCDH-PLD1 in conjunction with the luciferase reporter pGL3-empty vector, WT pGL3-IL7R-promoter, or pGL3-IL7R-promoter with EBS1 mutation. The results are expressed as fold-change relative to the corresponding cells transfected with the control vector after normalization of firefly luciferase activity according to Renilla luciferase activity. Experiments were independently repeated three times. Data are presented as the mean ± SD. Paired Student’s *t*-test was used for statistical analysis. *P < 0.05; **P < 0.01; ***P < 0.001; ns, not significant.

Next, we investigated the mechanism by which NPM1 and PLD1 mediate gemcitabine resistance. PLD1 affects the cellular location of NPM1, but not protein expression. Based on this finding, we inferred that NPM1 might function as a transcription factor. Recent studies have confirmed that NPM1 may act as a transcription factor^28^. Therefore, we utilized RNA-seq data on pLV-Vector and pLV-PLD1 PDAC cell lines to analyze differentially-expressed genes (DEGs). The top 50 DEGs between the control and PLD1 overexpression groups are presented as a heatmap based on log FC and P-values, with up- and down-regulated genes shown in red and blue, respectively (**Figure 5A**). These genes varied with PLD1 expression.

Further, we examined the level of DEG RNA expression in the EV, WT, NLM, and KRM cell lines. qPCR results revealed that the PLD1 downstream target genes (*IL7R* and *GSTT2*), and *NLRP1* were all significantly up-regulated in WT and KRM cell lines and down-regulated in the NLM cell line (**Figure 5B**). To confirm which downstream target genes of promoter regions bound NPM1, we surveyed the three gene promoter regions for potential NPM1 binding sites. It has been reported that NPM1 binds to a G-rich region in DNA with the repetitive sequence “TTAGGG,” which is also found on binding sites in the *IL7R, GSTT2*, and *NLRP1* promoter regions (**Figure 5C**)^27^. To further evaluate whether NPM1 binds directly to the *IL7R*, *GSTT2*, and *NLRP1* promoters, a ChIP assay was performed on the MIA PaCa-2 cell line (**Figure 5D**). Among the chromatin fractions pulled down by anti-NPM1 antibody, we only detected the IL7R promoter using PCR. We performed a ChIP-seq assay in the MIA PaCa-2 cell line to validate our screening using an anti-NPM1 antibody. The intersection of 17,920 genes was enriched by the NPM1 antibody in the ChIP-seq data and the genes expressed in the PLD1 overexpression cell line RNA-seq data. We assigned four genes in the Venn diagram, among which *IL7R* was enriched (**Figure 5E**). When the list was sorted from lowest-to-highest *P* values, *IL7R* was ranked first. Additionally, *IL7R* was pulled down twice in the CHIP-seq results (**Figure 5F**). These results showed that NPM1 binds at the promoter region of *IL7R* (**Supplementary Figure S6A, Supplementary Data File 2**). Finally, we performed re-ChIP to determine whether PLD1 also intersected with the *IL7R* promoter region (**Figure 5G, 5H**). In summary, PLD1 was shown to have a similar role to a co-transcription factor. In addition, we performed ChIP by anti-PLD1 in an NPM1 knockout cell line. When NPM1 was knocked out in cells, PLD1 did not combine with the IL7R promoter region (**Figure 5I**). Based on this finding, PLD1 requires NPM1 to combine with the *IL7R* promoter region.

### IL7R induced gemcitabine resistance *via* the Janus kinase 1 (JAK1)/activation of transcription (STAT5) signaling pathway

**Figure 6:**
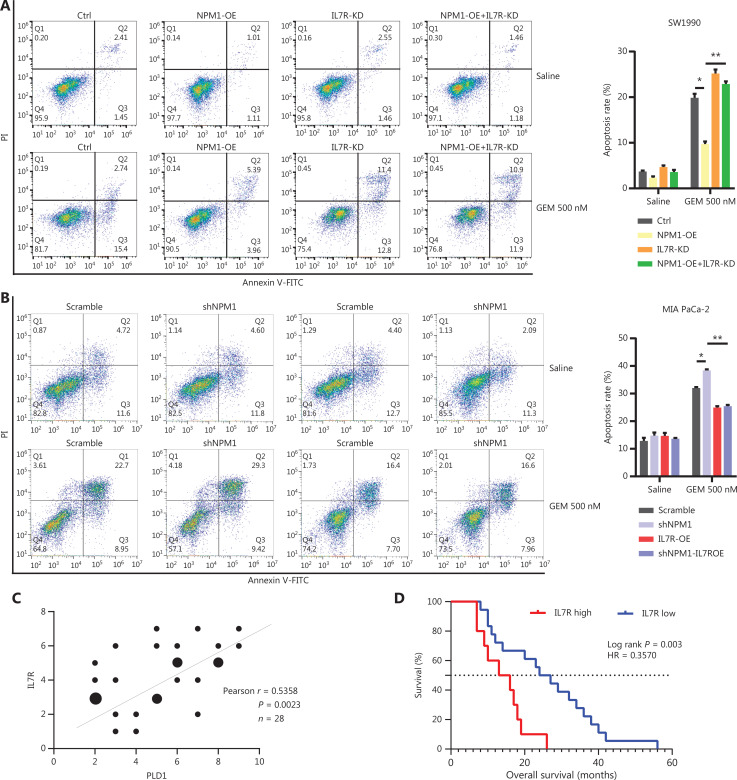

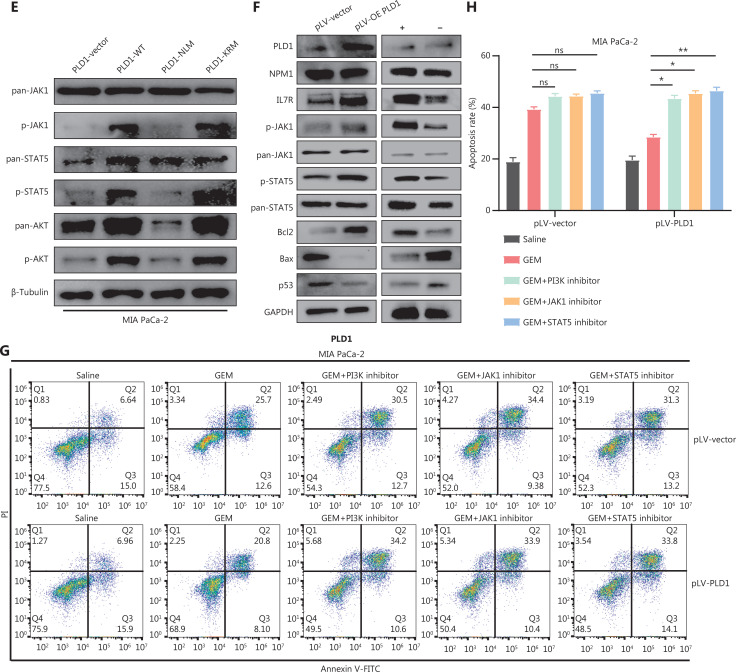
IL7R induced gemcitabine resistance *via* the JAK1/STAT5 signaling pathway. (A) Representative dot plots and statistical analysis of the frequency of CTRL (left), PLD1-OE (middle 1), IL7R-KD (middle 2), and PLD1-OE+IL7R-KD (right). (B) Representative dot plots and statistical analyses of the frequency of CTRL (left), IL7R-OE (middle 1), PLD1-KD (middle 2), and PLD1-KD+IL7R-OE (right). (C) Immunohistochemical (IHC) staining of tissues from 24 patients with PDAC using anti-PLD1 and -IL7R antibodies. Pearson correlation analysis between PLD1 and IL7R IHC scores. The bubble size represents the patient number with indicated IHC staining (from small-to-large; *n* = 1, *n* = 2, *n* = 3). (D) IHC results revealed that higher expression of IL7R in PDAC tissues was correlated with shorter OS (log-rank test). (E, F) The levels of p-JAK1, p-STAT5, and BCL-2 expression in MIA PaCa-2, EV, WT, nuclear localization signal mutation (NLM), and KRM cell lines using Western blot analysis. (G-H) MIA PaCa-2 cell line was treated with gemcitabine, a combination of gemcitabine and JAK1-IN-9, or STAT5-IN-1. Cytotoxicity was analyzed using flow cytometry. The data are expressed as the mean ± SEM from three independent experiments. *P < 0.05; **P < 0.01 (one-way ANOVA); ns, not significant.

To further determine whether the binding of NPM1 to the IL7R promoter upregulated gene transcription, we constructed two luciferase promoter vectors (pGL3-IL7R-WT and pGL3-IL7R-mutation). We transfected these vectors into HEK 293T and MIA PaCa-2 cell lines with or without a PLD1 and NPM1 expression vector (pCDH-PLD1) and utilized a pGL3-empty vector as the control. The dual luciferase reporter gene system determined that NPM1 and PLD1 overexpression significantly increased WT promoter activity (*P* < 0.01), but not in the pGL3-IL7R-mutation HEK 293T and MIA PaCa-2 cell lines (*P* < 0.01; **Figure 5I–5L**). These data indicate that NPM1 directly promotes IL7R transcription in PDAC by binding to the IL7R promoter. Having identified IL7R as the final downstream molecule, we performed blocking and reversion experiments to confirm the significant role that IL7R plays in gemcitabine resistance (**Figures 5E, 6A, 6B**). Also, the expression of IL7R protein was positively correlated with the nuclear expression of PLD1 (**Figure 6C, 6D**).

We analyzed PDAC samples in the Kyoto Encyclopedia of Genes and Genomes (KEGG database) to identify downstream mechanisms of IL7R upregulation. A previous study demonstrated the central regulatory role of IL7R in the lymphoid system^28^. The main signaling pathway that IL7R activates is the JAK1/STAT5 signaling pathway. We found that IL7R activation induced the phosphorylation of JAK1 and activated signal transducer and STAT5 proteins. Phosphorylated STAT5 dimerizes, translocates into the nucleus, and increases the expression of the anti-apoptotic BCL-2 protein^29–32^. To determine the key role of the IL7-IL7R/JAK1/STAT5 signaling pathway in PLD1-induced chemoresistance, we determined the phosphorylation levels at the different stages of the IL7-IL7R/JAK1/STAT5 signaling pathway. Upregulation of PLD1 led to an increase in the phosphorylation levels of JAK1 and STAT5. Conversely, PLD1 knockdown decreased the levels of phosphorylation of JAK1 and STAT5 (**Figure 6E, F**). These results indicate that PLD1 promotes JAK1/STAT5 signaling pathway activation. Additionally, we used an inhibitor of the JAK1/STAT5 signaling pathway to explore the effects of JAK1/STAT5 inhibition on PLD1-mediated gemcitabine resistance. We utilized JAK1-IN-9 and STAT5-IN-1 individually to treat SW1990 and MIA PaCa-2 cell lines. After 72 h of treatment with gemcitabine or saline, we performed CCK-8, colony formation, and apoptosis assays to further validate whether PLD1-induced gemcitabine resistance in PDAC is *via* the IL7R/JAK1/STAT5 signaling pathway. Remarkably, the effects induced by PLD1 were mostly abrogated by JAK1-IN-9 and STAT5-IN-1 (**Figure 6G, 6H**). Finally, we performed IHC to analyze the correlation of PLD1, IL17R, and pSTAT5 by serial section. The correlation between PLD1, IL17R, and pSTAT5 showed that nuclear PLD1 improved IL7R and pSTAT5 expression (**Supplementary Figure S6B**).

### Combination of PLD1 inhibition and gemcitabine potentially improves the survival of patients with PDAC

**Figure 7:**
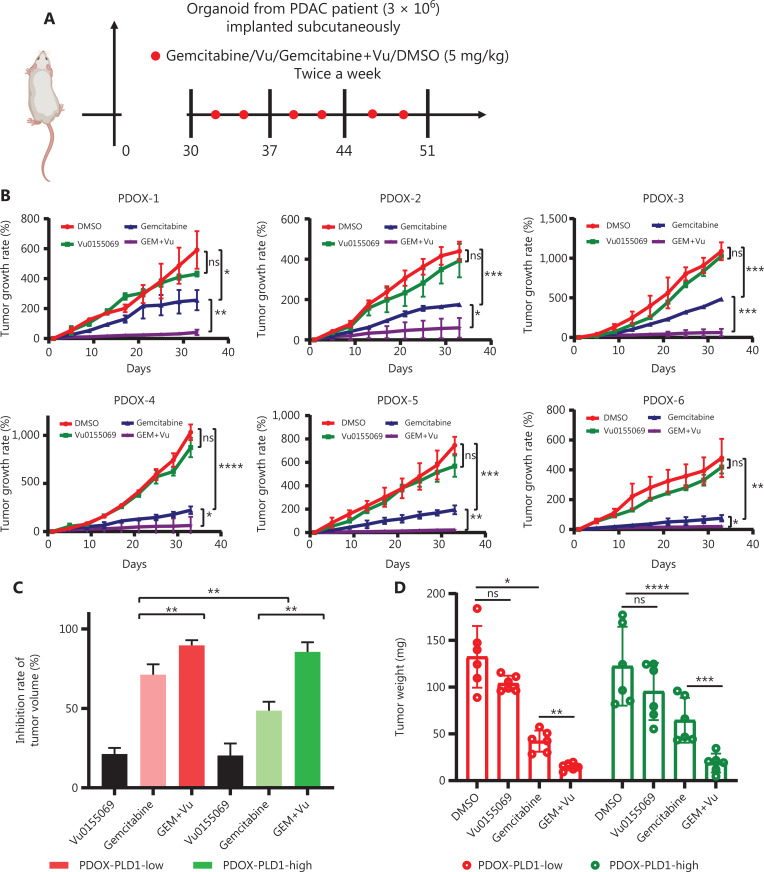

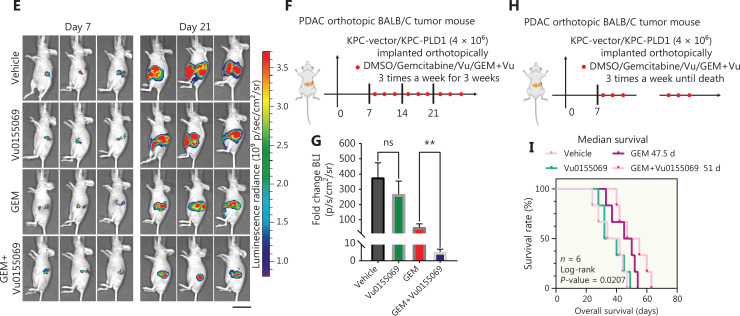
Potential roles for PLD1 as a marker for gemcitabine resistance therapy. (A) PLD1-high and -low expression groups of three PDAC organoids each were subcutaneously inoculated in nude mice on day 0. Tumor volume was measured every 3 d using calipers. Gemcitabine or Vu0155069 was injected intraperitoneally three times per week (red plots represent time points of drug administration). (B) Tumor growth curves of the four groups. (C) Statistical analysis of tumor growth inhibition rates from different groups. (D) Tumor weights at the end of the experiment. (E) Three representative bioluminescent images of the four groups on d 7 and 21 after tumor implantation. (F) C57BL/6 mice were orthotopically injected with 1 × 10^6^ luciferase-expressing KPC cells or luciferase-expressing cells in Matrigel. Gemcitabine or Vu0155069 was injected intraperitoneally three times per week (red plots represent time points of drug administration). (G) Statistical analysis of the BLI fold-change after treatment (BLI on d 21 to BLI on d 7). ***P < 0.001 by non-paired Student’s *t*-test (*n* = 9 per group). (H) C57BL/6 mice were orthotopically injected with 1 × 10^6^ luciferase-expressing KPC cells or luciferase-expressing cells in Matrigel. Gemcitabine or Vu0155069 was injected intraperitoneally three times per week until death (red plots represent time points of drug administration). (I) Kaplan–Meier survival curves with log-rank test for significance between gemcitabine treatment and gemcitabine plus Vu0155069 treatment. All mouse experiments were independently repeated three times using nine mice per experimental group in the orthotopic tumor model. Representative data are shown. Data are presented as the mean ± SD. *P < 0.05; **P < 0.01; ***P < 0.001; ****P < 0.0001; ns, not significant.

Previous studies have reported that Vu0155069 functions as a PLD1-selective inhibitor that induced a decrease in PLD1 expression and enzyme activity. Because tumors with high PLD1 expression exhibit relatively high gemcitabine resistance, we used subcutaneous patient-derived organoid (PDO)-based xenografts (PDOXs) and orthotopic mouse tumor models to determine whether Vu0155069 enhanced sensitivity to gemcitabine by reducing PLD1 activity. The PDOX models were used to mimic the effects of PLD1 inhibition on gemcitabine resistance in PDAC tissues. Orthotopic BALB/c tumor mouse models were used to elucidate the effect of PLD1 inhibition on the pancreatic microenvironment (**Figure 7A**).

We constructed PDO models using human PDAC tissues for PDOX experiments. Organoid models were classified into PLD1 high- and low-expression groups by IHC and immunoblot analysis. Similarly, we utilized the PDX models with the three highest and lowest levels of PLD1 expression for passaging. One month after inoculation, when the tumor size was ≥ 100 mm^3^, the two models were randomized and received saline, Vu0155069, gemcitabine, or gemcitabine plus Vu0155069 until the study endpoint. In the PDOX model, tumor growth was significantly inhibited by gemcitabine plus Vu0155069 compared with the single treatment groups (*P* = 0.017; **Figure 7B–7D**).

The KPC cell line was orthotopically injected into the pancreatic tissues of nude mice to create an orthotopic BALB/c tumor mouse model. The tumors were then randomly divided into Vu0155069, gemcitabine, decitabine plus Vu0155069, and control groups 7 d after inoculation. Tumor growth was evaluated by bioluminescent imaging (**Figure 7E, 7F**).

After receiving gemcitabine plus Vu0155069 treatment or the isotype control six times, bioluminescent imaging quantification demonstrated significantly reduced tumor growth in the combined treatment group compared with single treatment groups (**Figure 7G**). Notably, survival benefits were observed in the gemcitabine plus Vu0155069 group compared with the single treatment groups. (**Figure 7H, 7I**). Our study indicates that decreased PLD1 expression in PDAC may increase sensitivity to gemcitabine and that the level of PLD1 expression in PDAC is an indicator for gemcitabine treatment efficacy in clinical trials.

## Discussion

Gemcitabine is a front-line treatment for PDAC; however, gemcitabine resistance frequently arises during chemotherapy. The search for an efficient therapeutic target to inhibit gemcitabine resistance is ongoing. From unbiased genome-wide CRISPRa library screening coupled with transcriptome sequencing, we identified PLD1 as a very important gene responsible for gemcitabine resistance in PDAC. We demonstrated that Vu0155069, a PLD1 inhibitor, sensitized PDAC cells to gemcitabine treatment. Certainly, there are many other genes, equally important in gemcitabine resistance. In our study, we mainly focused on PLD1.

CRISPRa library screening enables researchers to identify genes contributing to a specific phenotype on a genome-wide scale. In this study we performed library screening using the GeCKO library, as previously described^33^. The read-depth confirmed the quality of our library screening, the number of missing sgRNAs, and sgRNA coverage in each group.

The effects of PLD1 on gemcitabine resistance were validated using three sgRNAs from the library and sequence-specific shRNA targeting PLD1. In addition, nude mouse models xenografted with MIA PaCa-2 pLV-vector and MIA PaCa-2 pLV-PLD1 cell lines suggested that PLD1 significantly increased gemcitabine resistance in PDAC cells *in vivo*. Similarly, clinical statistics showed that higher levels of PLD1 expression in tissues were correlated with poorer prognosis in patients with PDAC.

**Figure 8:**
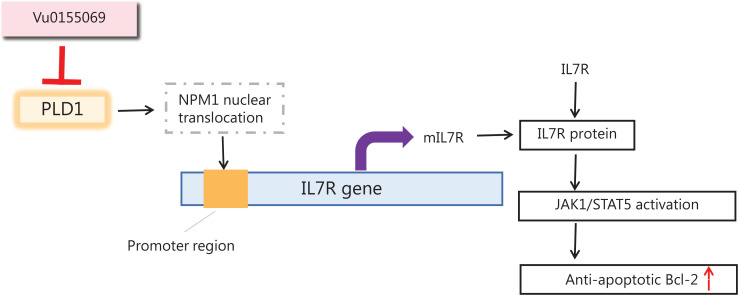
Schematic of PLD1 in gemcitabine resistance.

Additionally, we found that PLD1 has a significant role in gemcitabine resistance *via* PLD1 actions in the nucleus. PLD1 promoted chemotherapy resistance when combined with NPM1. Once bound to PLD1, NPM1 was translocated to the nucleus, where NPM1 bound to the promoter region of IL7R and acted as a transcription factor to upregulate IL7R expression. Activated IL7R induces the phosphorylation of JAK1 and STAT5. Phosphorylated STAT5 dimerizes and translocates into the nucleus, increasing the expression of the anti-apoptotic BCL-2 protein. By this mechanism, PLD1 mediated a remarkable enhancement in gemcitabine resistance in PDAC cell lines (**Figure 8**).

Past studies on PLD1 hinged on classical enzyme function. PLD1 is well-known to be a transphosphatidylase that hydrolyses PC to generate PA and choline dehydrocholate^13^. In this study we demonstrated that these two products of PLD1 do not elevate gemcitabine resistance. A recent study indicated that nuclear lipid metabolism genes might be used as novel prognostic genes in cancer. Indeed, our data suggest that the nuclear localization properties of PLD1 are the real drivers of gemcitabine resistance.

PX was traditionally regarded as the protein-protein interaction domain of PLD1. According to our results, PLD1 interacted with NPM1 *via* its PH domain^34,35^. When the PLD1 PH domain was deleted, both enzyme activity and normal PLD1 localization were inhibited. This finding suggests that the PLD1 nuclear localization sequence (NLS) may be located in the PH domain. It has been demonstrated that the IL7 cytokine combines with its ligand, IL7R, to improve lymphocyte homing^29^. Interestingly, certain studies in recent years have found that IL7R may be directly related to cancer; however, the function of IL7 and the IL7R may result in dual outcomes. IL7R exhibits antitumor effects in leukemia, prostate cancer, and gliomas, but exhibits tumor promotion in T-cell acute lymphoblastic leukemia and bladder cancer. In previous studies, the exact effects of IL7 on PDAC were unclear^36^. Our results indicate that IL7R may prevent apoptosis by regulating the BCL2 gene family in PDAC.

IL7/IL7R have long been considered essential molecules for maintaining the stability of T-cell development^37^. In previous studies analysis of the thymus in IL7-deficient mice showed that the T cell development stagnated at the triple-negative stage (CD32^−^, CD42^−^, and CD82^−^). These cells showed an increased rate of spontaneous apoptosis, decreased expression levels of the anti-apoptotic BCL-2 protein, and increased levels of Bax, the pro-apoptotic BCL-2 homolog^38^; however, when these cells were treated with IL7, BCL-2 expression was upregulated, inhibiting apoptosis. In mice with an IL7/IL7R axis arrest, the OE of BCL-2 largely restored T cell development. This finding suggests that the IL7/IL7R signaling axis is regulated by BCL-2^39,40^.

Many studies on hematologic diseases prove that the IL7/IL7R axis is also an important factor in the development of leukemia. *In vitro* studies have shown that the IL7R in t-line acute lymphoblastic leukemia (T-ALL) mediates T-ALL cell proliferation and survival after binding to IL7 secreted by bone marrow and thymic stromal cells^41–43^. Greater than 70% of the patients with T-ALL have IL7R-positive blast cells, and disease status is closely associated with IL7Ra expression. In previous studies, IL-7, IL-4, and IL-2 were confirmed to be growth factors in pediatric T-ALL^13–15^; however, other studies have demonstrated that upregulation of BCL-2 expression after IL7 binding to the IL7R is an efficient inhibitor of spontaneous apoptosis in T-ALL cells^44,45^.

The IL7R is well-known to be a receptor bound by IL7Ra to IL2RG. In hematologic diseases, IL7Ra expression is a key factor affecting apoptosis, independent of IL2RG, another subunit of IL7R receptor^46^. In this study we found that IL7Ra expression in the population was greatly increased, and the proportion of apoptosis in T-ALL-like cells decreased significantly when IL7Ra expression was increase; however, IL2RG was expressed at similar levels in human populations and independent of leukemic cell responsiveness to IL7. The IL7R in PDAC cells upregulated by PLD1 and NPM1 increased anti-apoptotic molecules, such as BCL-2, thus enhancing gemcitabine resistance in solid tumor cells.

Our study had a notable limitation. Specifically, it is still unknown in what forms the IL7R exists in PDAC. In leukemic cells, there are four mutant forms of the IL7 receptor^47–50^. In this study we did not verify which isoforms of the IL7 receptor were expressed that induce the upregulation of Bcl2 anti-apoptotic molecules in pancreatic tumor cells.

Vu0155069 inhibits the enzyme activity and expression of PLD1, and have also been shown to have inflammasome inhibition function in recent studies^51^. The regulation of inflammasome participates in numerous inflammatory diseases. Vu0155069 inhibits caspase-1 to reduce the production of inflammasome. Thus, IL-1β produced by an inflammasome would be significantly reduced. The current studies confirmed that Crohn’s disease and arthrolithiasis are both interrelated to inflammasome activity. Indeed, Vu0155069 can effect the process of these inflammatory diseases. In addition, IL-1β also has an important role in the pathologic course of sepsis. Therefore, PLD1 participates in bacterial and viral infectious diseases; however, the mechanism underlying the Vu0155069 effect on caspase-1 activity is unclear.

Recent studies have confirmed that fatty acid metabolism also participates in the development of multiple cancers^25^. Several studies have reported expression of PLD1 in various human cancers, such as breast, kidney, gastric, thyroid, and colorectal cancers. PLD1 has been well-established as a key regulator of the proliferation and survival of many different cancer cells. As mentioned earlier, PLD1 catalyzes the hydrolysis of PC into PA and choline^52^. PA, as a key lipid second messenger, regulates cell cycle and proliferation-related proteins. As a serine-threonine kinase, m-TOR is a key downstream target of PA that regulates cell metabolism, proliferation, and survival. Rapamycin can affect the survival and growth of tumor cells by disrupting the stability of m-TOR^53^. Similarly, Vu155069 is a selective PLD1 inhibitor; however, Vul55069 may also have PLD1-independent effects, such as fatty acid synthesis inhibition.

In our study we found that VU0155069 reverses gemcitabine resistance by inhibiting PLD1 nuclear migration. The combined VU0155069-gemcitabine treatment cytotoxicity is probably related to gemcitabine cytotoxicity, avoidance of gemcitabine resistance, and independent VU0155069 antitumoral effects. This viewpoint needs further verification.

## Data Availability

All data relevant to the study are included in the article or uploaded as supplementary information.

## References

[1] Siegel RL, Miller KD, Jemal A (2019). Cancer statistics. 2019. CA Cancer J Clin.

[2] Rawla P, Sunkara T, Gaduputi V (2019). Epidemiology of pancreatic cancer: global trends, etiology and risk factors. World J Oncol.

[3] Maomao C, He L, Dianqin S, Siyi H, Xinxin Y, Fan Y (2022). Current cancer burden in China: epidemiology, etiology, and prevention. Cancer Biol Med.

[4] Macarulla T, Fernández T, Gallardo ME, Hernando O, López AM, Hidalgo M (2017). Adjuvant treatment for pancreatic ductal carcinoma. Clin Transl Oncol.

[5] Kamisawa T, Wood LD, Itoi T, Takaori K (2016). Pancreatic cancer. Lancet.

[6] Ireland L, Santos A, Ahmed MS, Rainer C, Nielsen SR, Quaranta V (2016). Chemoresistance in pancreatic cancer is driven by stroma-derived insulin-like growth factors. Cancer Res.

[7] Shukla SK, Purohit V, Mehla K, Gunda V, Chaika NV, Vernucci E (2017). MUC1 and HIF-1alpha signaling crosstalk induces anabolic glucose metabolism to impart gemcitabine resistance to pancreatic cancer.. Cancer Cell.

[8] Dauer P, Nomura A, Saluja A, Banerjee S (2017). Microenvironment in determining chemo-resistance in pancreatic cancer: Neighborhood matters. Pancreatology.

[9] Hoos WA, James PM, Rahib L, Talley AW, Fleshman JM (2013). Matrisian LM. Pancreatic cancer clinical trials and accrual in the United States. J Clin Oncol.

[10] Mini E, Nobili S, Caciagli B, Landini I, Mazzei T (2006). Cellular pharmacology of gemcitabine. Ann Oncol.

[11] Binenbaum Y, Na’ara S, Gil Z (2015). Gemcitabine resistance in pancreatic ductal adenocarcinoma. Drug Resist Updat.

[12] Chen M, Mao A, Xu M, Weng Q, Mao J, Ji J (2019). CRISPR-Cas9 for cancer therapy: opportunities and challenges. Cancer Lett.

[13] Zhang F, Wen Y, Guo X (2014). CRISPR/Cas9 for genome editing: progress, implications and challenges. Hum Mol Genet.

[14] Bowling FZ, Frohman MA, Airola MV (2021). Structure and regulation of human phospholipase D. Adv Biol Regul.

[15] Barber CN, Huganir RL, Raben DM (2018). Phosphatidic acid-producing enzymes regulating the synaptic vesicle cycle: role for PLD?. Adv Biol Regul.

[16] Gomez-Cambronero J, Fite K, Miller TE (2018). How miRs and mRNA deadenylases could post-transcriptionally regulate expression of tumor-promoting protein PLD. Adv Biol Regul.

[17] Noble AR, Maitland NJ, Berney DM, Rumsby MG (2018). Phospholipase D inhibitors reduce human prostate cancer cell proliferation and colony formation. Br J Cancer.

[18] Shi Y, Liu M, Huang Y, Zhang J, Yin L (2020). Promotion of cell autophagy and apoptosis in cervical cancer by inhibition of long noncoding RNA LINC00511 via transcription factor RXRA-regulated PLD1. J Cell Physiol.

[19] Zhou W, Shi K, Ji L, Wu R, Chen Y, Tu H (2018). Inhibition of phospholipase D1 mRNA expression slows down the proliferation rate of prostate cancer cells that have transited to androgen independence. J Cancer.

[20] Yao B, Li Y, Chen T, Niu Y, Wang Y, Yang Y (2021). Hypoxia-induced cofilin 1 promotes hepatocellular carcinoma progression by regulating the PLD1/AKT pathway. Clin Transl Med.

[21] Kang DW, Min G, Park DY, Hong KW, Min DS (2010). Rebamipide-induced downregulation of phospholipase D inhibits inflammation and proliferation in gastric cancer cells. Exp Mol Med.

[22] Son JC, Kang DW, Yang KM, Choi K-Y, Son TG, Min DS (2013). Phospholipase D inhibitor enhances radiosensitivity of breast cancer cells. Exp Mol Med.

[23] Jones D, Morgan C, Cockcroft S (1999). Phospholipase D and membrane traffic. Potential roles in regulated exocytosis, membrane delivery and vesicle budding. Biochim Biophys Acta.

[24] Lee SK, Kim YS, Bae GH, Lee HY, Bae Y-S (2019). VU0155069 inhibits inflammasome activation independent of phospholipase D1 activity. Sci Rep.

[25] Lee TJ, Kim YH, Min do S, Park JW, Kwon TK (2006). Se-methylselenocysteine enhances PMA-mediated CD11c expression via phospholipase D1 activation in U937 cells. Immunobiology.

[26] Siddiqi AR, Srajer GE, Leslie CC (2000). Regulation of human PLD1 and PLD2 by calcium and protein kinase C. Biochim Biophys Acta.

[27] Zhang X, Huang C, Yuan Y, Jin S, Zhao J, Zhang W (2022). FOXM1-mediated activation of phospholipase D1 promotes lipid droplet accumulation and reduces ROS to support paclitaxel resistance in metastatic cancer cells. Free Radic Biol Med.

[28] Jang YH, Min do S (2011). Nuclear localization of phospholipase D1 mediates the activation of nuclear protein kinase C(alpha) and extracellular signal-regulated kinase signaling pathways. J Biol Chem.

[29] Qin G, Wang X, Ye S, Li Y, Chen M, Wang S (2020). NPM1 upregulates the transcription of PD-L1 and suppresses T cell activity in triple-negative breast cancer. Nat Commun.

[30] Barata JT, Durum SK, Seddon B (2019). Flip the coin: Il-7 and IL-7R in health and disease. Nat Immunol.

[31] Follini E, Marchesini M, Roti G (2019). Strategies to overcome resistance mechanisms in T-cell acute lymphoblastic leukemia. Int J Mol Sci.

[32] Shi L, Xu Z, Yang Q, Huang Y, Gong Y, Wang F (2019). IL-7-Mediated IL-7R-JAK3/STAT5 signalling pathway contributes to chemotherapeutic sensitivity in non-small-cell lung cancer. Cell Prolif.

[33] Lin J, Zhu Z, Xiao H, Wakefield MR, Ding VA, Bai Q (2017). The role of IL-7 in immunity and cancer. Anticancer Res.

[34] Wei L, Lee D, Law CT, Zhang MS, Shen J, Chin DW (2019). Genome-wide CRISPR/Cas9 library screening identified PHGDH as a critical driver for sorafenib resistance in HCC. Nat Commun.

[35] Stahelin RV, Ananthanarayanan B, Blatner NR, Singh S, Bruzik KS, Murray D (2004). Mechanism of membrane binding of the phospholipase D1 PX domain. J Biol Chem.

[36] Hodgkin MN, Masson MR, Powner D, Saqib KM, Ponting CP, Wakelam MJ (2000). Phospholipase D regulation and localisation is dependent upon a phosphatidylinositol 4,5-biphosphate-specific PH domain. Curr Biol.

[37] Bardelli V, Arniani S, Pierini V, Di Giacomo D, Pierini T, Gorello P (2021). T-cell acute lymphoblastic leukemia: biomarkers and their clinical usefulness. Genes (Basel).

[38] Dias S, Silva H, Cumano A, Vieira P (2005). Interleukin-7 is necessary to maintain the B cell potential in common lymphoid progenitors. J Exp Med.

[39] Rathmell JC, Farkash EA, Gao W, Thompson CB (2001). IL-7 enhances the survival and maintains the size of naive T cells. J Immunol.

[40] Ribeiro D, Melão A, van Boxtel R, Santos CI, Silva A, Silva MC (2018). STAT5 is essential for IL-7-mediated viability, growth, and proliferation of T-cell acute lymphoblastic leukemia cells. Blood Adv.

[41] Zenatti PP, Ribeiro D, Li W, Zuurbier L, Silva MC, Paganin M (2011). Oncogenic IL7R gain-of-function mutations in childhood T-cell acute lymphoblastic leukemia. Nat Genet.

[42] Shochat C, Tal N, Bandapalli OR, Palmi C, Ganmore I, te Kronnie G (2011). Gain-of-function mutations in interleukin-7 receptor-α (IL7R) in childhood acute lymphoblastic leukemias. J Exp Med.

[43] Zhang J, Ding L, Holmfeldt L, Wu G, Heatley SL, Payne-Turner D (2012). The genetic basis of early T-cell precursor acute lymphoblastic leukaemia. Nature.

[44] Maeurer MJ, Walter W, Martin D, Zitvogel L, Elder E, Storkus W (1997). Interleukin-7 (IL-7) in colorectal cancer: Il-7 is produced by tissues from colorectal cancer and promotes preferential expansion of tumour infiltrating lymphocytes. Scand J Immunol.

[45] Qu H, Zou Z, Pan Z, Zhang T, Deng N, Chen G (2016). IL-7/IL-7 receptor axis stimulates prostate cancer cell invasion and migration via AKT/NF-κB pathway. Int Immunopharmacol.

[46] Kim GY, Hong C, Park JH (2011). Seeing is believing: illuminating the source of in vivo interleukin-7. Immune Netw.

[47] Yokoyama K, Yokoyama N, Izawa K, Kotani A, Harashima A, Hozumi K (2013). In vivo leukemogenic potential of an interleukin 7 receptor α chain mutant in hematopoietic stem and progenitor cells. Blood.

[48] Park JH, Waickman AT, Reynolds J, Castro M, Molina-París C (2019). IL7 receptor signaling in T cells: a mathematical modeling perspective. Wiley Interdiscip Rev Syst Biol Med.

[49] Verstraete K, Peelman F, Braun H, Lopez J, Van Rompaey D, Dansercoer A (2017). Structure and antagonism of the receptor complex mediated by human TSLP in allergy and asthma. Nat Commun.

[50] Omraninava M, Mehranfar S, Vahedi P, Razi B, Imani D, Aslani S (2021). Association between IL7 Receptor Alpha (Il7ra) gene rs6897932 polymorphism and the risk of Multiple Sclerosis: a meta-regression and meta-analysis. Mult Scler Relat Disord.

[51] Marković I, Savvides SN (2020). Modulation of signaling mediated by TSLP and IL-7 in inflammation, autoimmune diseases, and cancer. Front Immunol.

[52] Munir R, Lisec J, Swinnen JV, Zaidi N (2022). Too complex to fail? Targeting fatty acid metabolism for cancer therapy. Prog Lipid Res.

[53] Wang H, Wu S, Yan Wang Y, Tang B (2022). Rosiglitazone disrupts pancreatic ductal adenocarcinoma progression by activating the tumor suppressor ESE3/EHF. Cancer Biol Med.

