# Supplementary material for: Nuclear PLD1 combined with NPM1 induces gemcitabine resistance through tumorigenic IL7R in pancreatic adenocarcinoma
Source: Cancer Biol Med. 2023 Jun 27;20(8):599–626. doi: 10.20892/j.issn.2095-3941.2023.0039 (PMC10476466; doi:10.20892/j.issn.2095-3941.2023.0039)
Supplement: Supplementary file 1 [file cbm-20-599-s001.zip › cbm-20-599-s003.docx]

**Document Supplementary1（Doc S1）**

Materials and methods

**(1)Preparing, passaging, and freezing human organoids**

Primary human PDAC organoids were established from fresh, surgically resected human PDAC tissues from different patients with PDAC. The tumor tissue was masticated into small fragments (approximately 1 mm^3^) and digested with Collagenase Crude XI (#C9407-1G, Sigma-Aldrich) in a rotating incubator at 37 °C at 35 rpm for 15 min, twice. After each digestion, we used the Advanced DMEM/F-12 medium (#12634010, Thermo Fisher) with 0.1% bovine serum albumin (BSA; h+++B) to neutralize the collagenase and collected the supernatant after centrifugation at 300 × g for 5 min at 4 °C. We removed the supernatant and resuspended the cell fraction in growth factor reduced Matrigel (#356231, Corning) for 30 min at 37 °C and cultured the cells in human complete medium (Advanced DMEM/F-12 medium [#12634010, Thermo Fisher] supplemented with HEPES [#15630080, Thermo Fisher], Glutamax [#35050061, Thermo Fisher], penicillin/streptomycin [#15070063, Thermo Fisher], B27 [#17504044, Thermo Fisher], Primocin [#ant-pm-2, InvivoGen], N-Acetylcysteine [#A9165-5G, Sigma-Aldrich], Wnt3a-conditioned medium [50% v/v, L Wnt-3A Cells, CRL-2647, ATCC], RSPO1-conditioned medium [10% v/v, Calvin Kuo], Recombinant Murine Noggin (#250-38, Peprotech), Recombinant Human FGF-10 (#100-26, Peprotech), Gastrin I (human) [#3006, Tocris], Recombinant Human FGF-10 [#100-26, Peprotech], Nicotinamide [#N0636-100G, Sigma-Aldrich], A83-01 [#2939, Tocris], Prostaglandin E2 [#2296 //10, TOCRIS/R&D Systems] and Y-27632 [#Y0503, Sigma-Aldrich]).

The complete human medium for PDAC organoid culture was changed every 2nd day. The small spheres structures of the primary PDAC organoids were visually inspected after 2–3 weeks of culture. Upon the attainment of dense culture, the organoids were digested into small fragments using the TrypLE™ Express Enzyme (#12604021, Thermo Fisher) and transferred to a fresh matrix in the human complete medium.

All organoid cultures were dissociated and mixed with 100% FBS and frozen to prepare frozen stocks, following standard procedures. When required, the cultures were thawed using standard thawing procedures and cultured as described above.

**(2)Western blot analysis**

Cell, tissue, or organoid lysates were prepared in protein lysate supplemented with a protease inhibitor (#4693116001, Sigma-Aldrich) cocktail. Protein concentration was measured by the BCA protein assay (#23227, Thermo Fisher). Equal amounts of protein were diluted in Ultrapure water and 4× LDS Sample Buffer 5 (#M00676, Genescript) for 10 min at 100 °C. Samples were loaded on 10% polyacrylamide gel run for 45 min at 80 V and 1.5 h at 120 V and then transferred for 2 h at a constant electric current of 0.26 A to a polyvinylidene fluoride membrane (0.45 μm). Membranes were blocked with 3% BSA for 1.5 h at 20–30 °C. Incubation with primary antibodies was done overnight at 4 °C. The membranes were washed 3 × for 10 min in TBS-T (Tris Buffered Saline + 0.05% Tween20) the following day. The secondary antibodies were applied to the membrane and incubated for 2 h at 20–30 °C. After another three washes with TBS-T, signal detection was achieved by incubating the membrane with the Immobilon Western HRP Substrate (#WBKLS0500, Millipore). Target proteins were detected with the following primary antibodies: anti-PLD1 (#3832s, Cell Signaling Technology, 1:1000), anti-NPM1 (#10306-1-AP, Proteintech, 1:1000), anti–IL7R (#ab95024, Abcam, 1:1000), anti-GAPDH (#ab8245, Abcam, 1:5000), and anti–β-tubulin (#ab210797, Abcam, 1:5000). The secondary antibodies were goat anti-rabbit (#7074s, Cell Signaling Technology) or mouse antibody (#7076s, Cell Signaling Technology) at a 1:5000 dilution.

**(3)Subcutaneous and Orthotopic Xenograft Model**

Six-week-old female BALB/c nude mice were purchased from SPF Biotechnology Company (Beijing, China). All mice in this study were maintained in specific pathogen-free conditions, and animal experiment procedures were approved by the Ethics Committee of Tianjin Medical University Cancer Institute and Hospital. The experiments were performed in compliance with the principles and procedures of the NIH Guide for the Care and Use of Laboratory Animals. Tumor cells were harvested by trypsinization, washed in PBS, and resuspended at 1 × 10^7^ cells/mL in PBS. A total of 1 × 10^6^ and 4 × 10^4^ cells were subcutaneously and orthotopically injected into the mice, respectively. Tumor size and the weight of the mice were measured thrice weekly. After one or two weeks, when the size of the tumor was approximately 100 mm^3^, gemcitabine (15 mg/kg), Vu0155069 (15 mg/kg), gemcitabine+Vu, and 10% dimethyl sulfoxide (DMSO) were intraperitoneally injected into the corresponding mice thrice weekly for three weeks. For the orthotopic tumor model, tumor size was detected by *in vivo* bioluminescence imaging.

**(4)PDX (patient-derived xenograft) and PDOX (patient-derived organoid xenograft) mice models**

Patient recruitment and enrolment were completed through the Tianjin Medical University Cancer Institute and Hospital, and patients provided written informed consent authorizing the collection and use of tumor tissue and clinical data for study purposes. Pancreatic tumors were collected from the operating room during surgical resection.

For the PDX mice models, primary tumor specimens were engrafted as soon as possible into female NSG (NOD SCID gamma) mice purchased from Biocytogen (Beijing, China) on the same day as primary resection to establish the PDX mice. When the tumor size reached approximately 300 mm^3^, the tumor was stripped from the NSG mice and minced into 1-mm^3^ fragments for implantation. The tumor fragment was subcutaneously inoculated into the flank region of other NSG mice, and the incision was closed with a surgical suture.

For the PDOX mice models, primary tumors were first used to construct the organoid model *in vitro* (method described above). To generate the PDO-derived xenografts, organoids were harvested from 6-well plates, mechanically dissociated, resuspended in 100 μL Matrigel, and subcutaneously injected into 6-week-old female BALB/c nude mice purchased from SPF Biotechnology Company (Beijing, China).

After approximately one month, when the PDX/PDOX mice tumor size reached 100 mm^3^, the drugs were injected into the corresponding mice as described above. The size of the tumor and mice weight were measured thrice weekly. The PDX/PDOX tumors were ready for harvest three weeks after drug injection. The tumor was stripped and cut into fragments for several purposes, flash frozen, and paraffin-embedded for histopathologic analysis.

**(5)Construction of drug-resistant and drug-sensitive cell lines-GR and GS**

First, take MIA PaCa-2 cells in the logarithmic growth phase (confluence 60% -80%) to divided into two 10-cm dishes and were treated with DMEM and low starting concentration (100 nM gemcitabine) for 24h. And then discard the culture medium, PBS wash twice, replace DMEM.After the cells resume growth, digest and passage cells and keep culture cells at low concentration (100nM) for 24h in one dish. after the cells proliferate to normal morphology, the drug impact was repeated with gradually increase the drug dose, each concentration shock 6-8 times. After the cells grow stably at this concentration, continue to increase the drug concentration, and the drug increases the concentration successively. Drug induction lasted for 6 – 8 months until the cells were able to grow stably in concentrations of the drug.
