# Supplementary material for: Nuclear PLD1 combined with NPM1 induces gemcitabine resistance through tumorigenic IL7R in pancreatic adenocarcinoma
Source: Cancer Biol Med. 2023 Jun 27;20(8):599–626. doi: 10.20892/j.issn.2095-3941.2023.0039 (PMC10476466; doi:10.20892/j.issn.2095-3941.2023.0039)
Supplement: Supplementary file 1 [file cbm-20-599-s001.zip › cbm-20-599-s010.docx]

***Supplementary Information***

**Supplementary Figures**


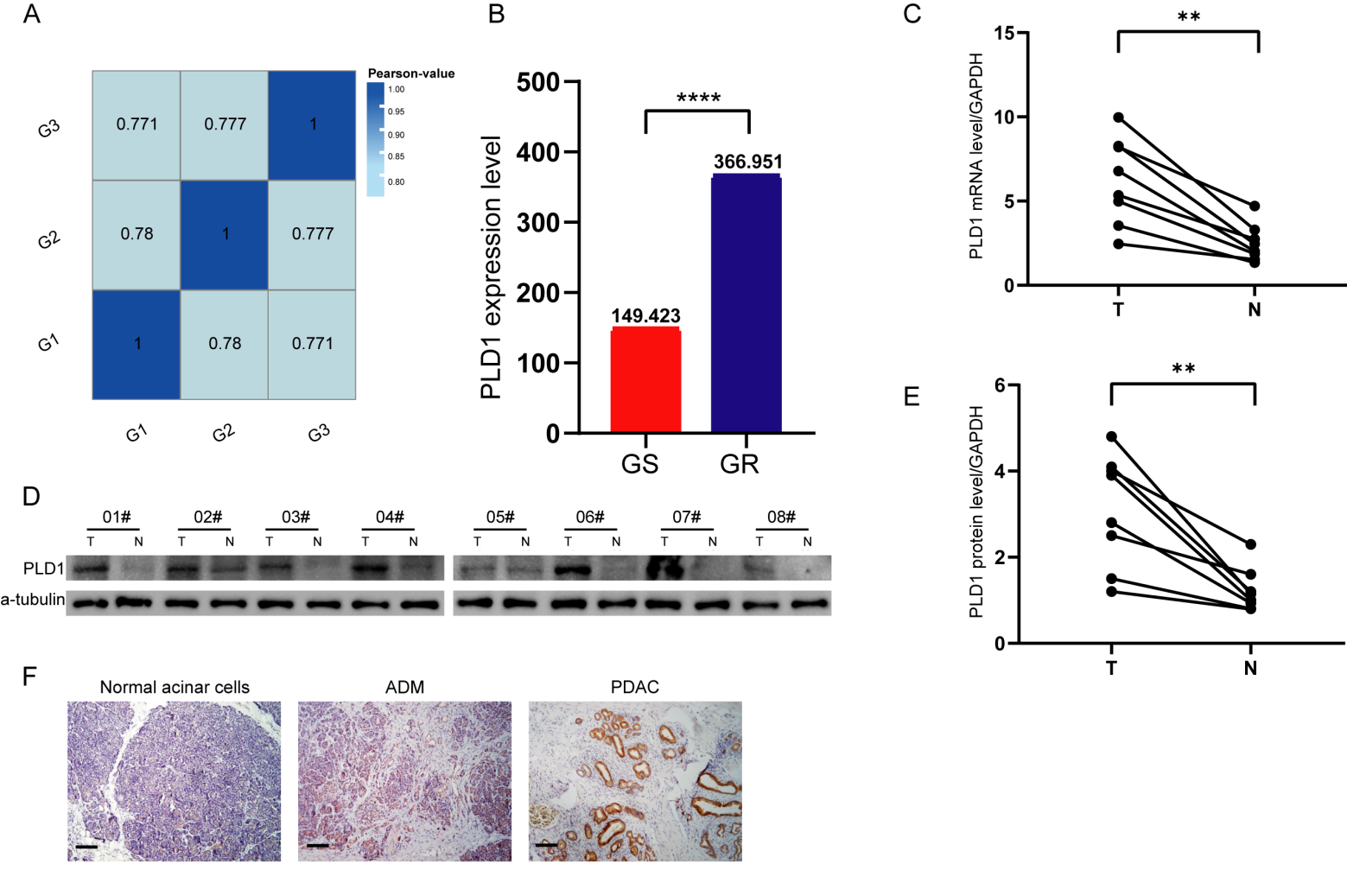


**Figure S1.** (**A**) The reproducibility of three independent replicate samples genome-wide CRISPRa/ dCas9 transcriptional activation library. (**B**) PLD1 mRNA expression level in the indicated cell lines. qPCR was performed to detect the mRNA differential multiple in GS and GR. (**C–E**) Western blot and qPCR analysis of PLD1 levels in eight paired PDAC tumorous and adjacent normal pancreatic tissues (T, tumour tissues; N, normal tissues). The corresponding statistics are displayed in the line chart. (**F**) Representative IHC images of PLD1 protein expression using human PDAC, ADM and normal acinar tissue sections.

**Figure S2.** (**A, B**) The RNA and protein expression levels of CRISPRa sgRNA OE cell lines by Western blotting and PCR. (**C**) Cell viability was examined by CCK-8 assay after 72 h treatment with gemcitabine (*n* = 3, per group). PLD1 significantly increased gemcitabine resistance in BxPC-3. (**D**) Cell viability was performed by CCK-8 assay after 72 h gemcitabine treatment (*n* = 3, per group). PLD1 significantly increased gemcitabine resistance in MIA PaCa-2 sgRNA overexpression cell lines. (**E**) Representative images and quantification of colony formation in BxPC-3 cell lines that were treated for 72 h with gemcitabine or saline. (**F–H**) Flow cytometry was performed to detect the apoptosis rates of cell lines treated with 500 nM gemcitabine or saline for 72 h in PLD1 overexpression or knock down cell lines. The corresponding statistics are presented in the histogram.


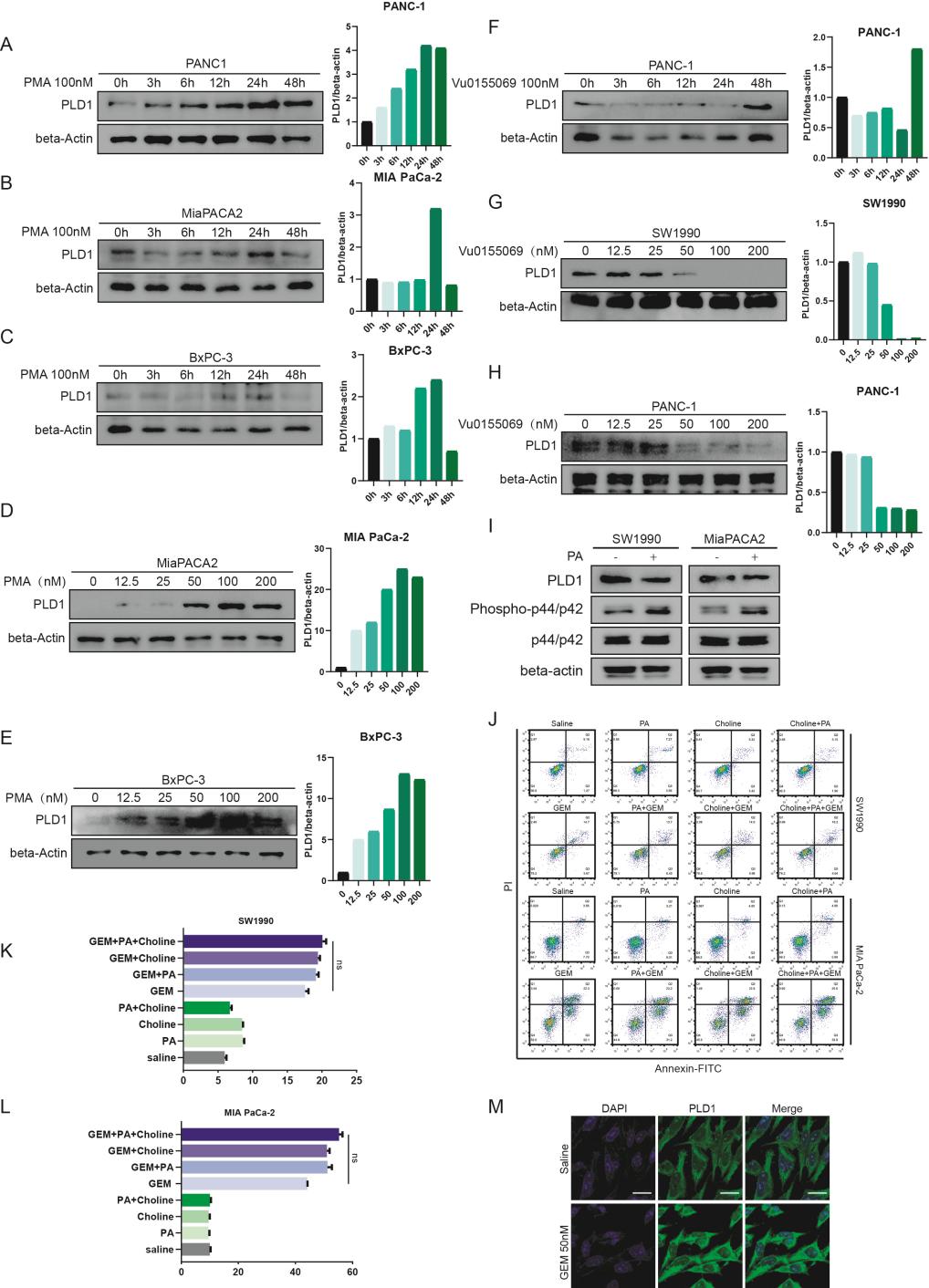


**Figure S3.** (**A–C**) WB was performed after PMA treated the indicated cell lines with corresponding time to display the change of PLD1. The time gradient is 0 h, 3 h, 6 h, 12 h, 24 h, 48 h. (**D, E**) WB was performed after the indicated cell lines were treated in (0, 12.5, 25, 50, 100, 200 nM) concentration gradient to find the change of PLD1. (**F–H**) WB was performed after Vu0155069 treated the indicated cell lines with corresponding time gradient and concentration gradient. (**I**) SW1990 and MIA PaCa-2 were treated by 100 nM PA for WB to reveal the change of pathway.(**J-L**) Flow cytometry was performed to detect the apoptosis rates of the cell lines pre-treated with PA, choline, or PA plus choline treated with 500 nM gemcitabine or saline for 72 h. The corresponding statistics are presented in the histogram. (**M**)Pretreated MIA PaCa-2 cell line with 50nM gemcitabine or saline.In the IF, we found that gemcitabine could increase the PLD1 expression in whole cell.

**Figure S4.** (**A–D**) Nucleocytoplasmic separation western blotting was performed to measure intranuclear and extranuclear PLD1 distribution after pre-treatment with choline and Vu0155069. (**E**) The schema chart of nuclear localization sequence (NLS) mutation (NLM) PLD1 cell line.(F) The protein expression levels of Ctrl, PLD1-OE, NPM1-KD and PLD1-OE NPM1-KD cell lines were detected by Western blotting.

**Figure S5.** (**A**) The schema chart of domain-truncated PLD1. (**B**) A group of PLD1 deletion constructs CO-IP with NPM1 to find the real domain that binds with it. (**C**) The schema chart of domain-truncated NPM1. (**D**) A set of NPM1 deletion constructs performed CO-IP to reveal that C-terminal△3 was sufficient to interact with PLD1.(E) The protein expression levels of Ctrl, NPM1-OE, IL7R-KD and IL7R-OE NPM1-KD cell lines were detected by Western blotting.


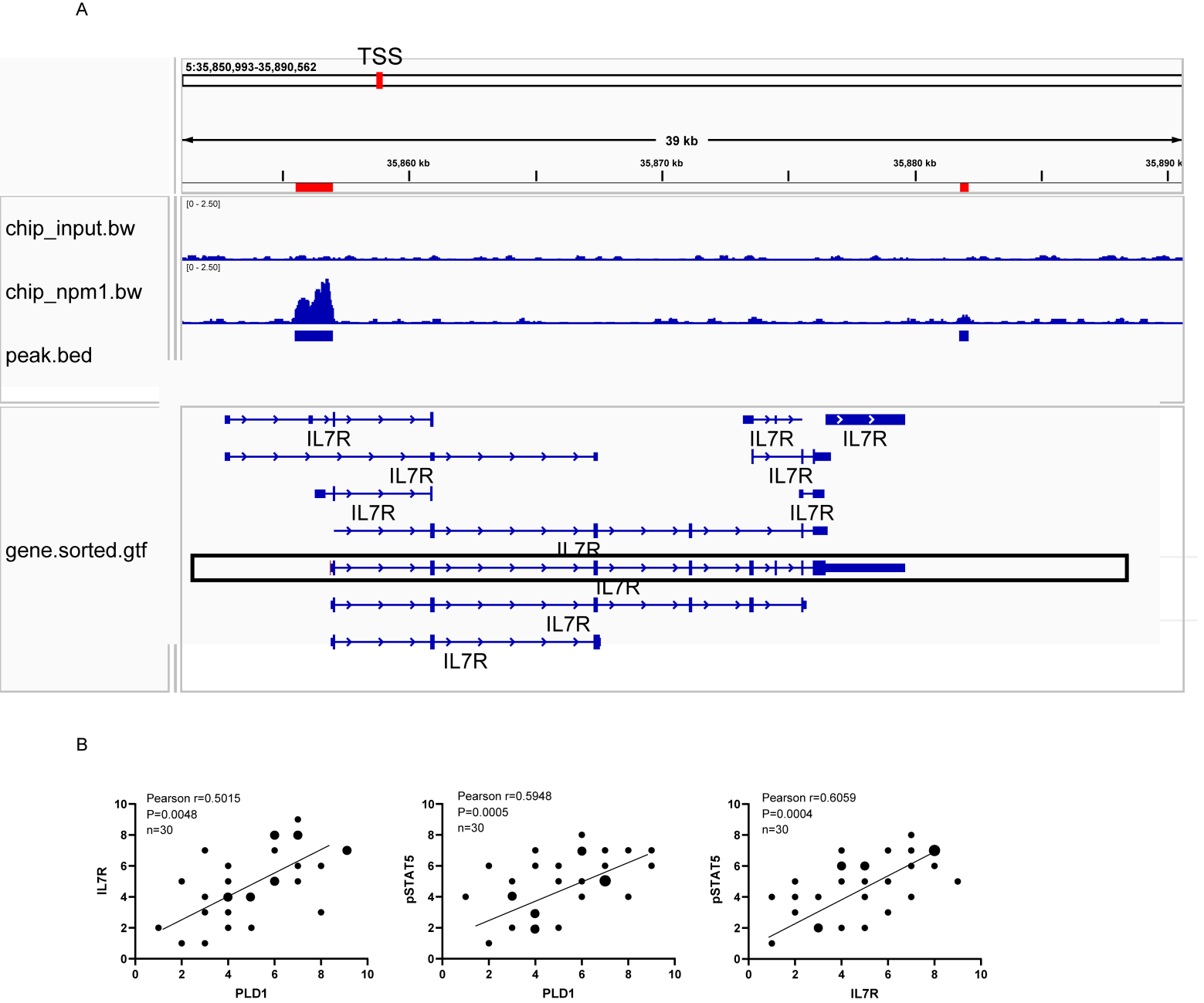


**Figure S6. (A)**The CHIP-seq peak plots reveal that the NPM1 binding position is on IL7R gene.(B) Immunohistochemical (IHC) staining of tissues from 30 patients with PDAC with anti-PLD1, anti-IL7R and pSTAT5 antibodies. Pearson correlation analysis the two protein from PLD1 IL7R and pSTAT5 IHC scores.The bubble size represents the patient number with indicated IHC staining. （from small to large, n=1, n=2, n=3）
