# Supplementary figures and images for: Nuclear PLD1 combined with NPM1 induces gemcitabine resistance through tumorigenic IL7R in pancreatic adenocarcinoma
Source: Cancer Biol Med. 2023 Jun 27;20(8):599–626. doi: 10.20892/j.issn.2095-3941.2023.0039 (PMC10476466; doi:10.20892/j.issn.2095-3941.2023.0039)

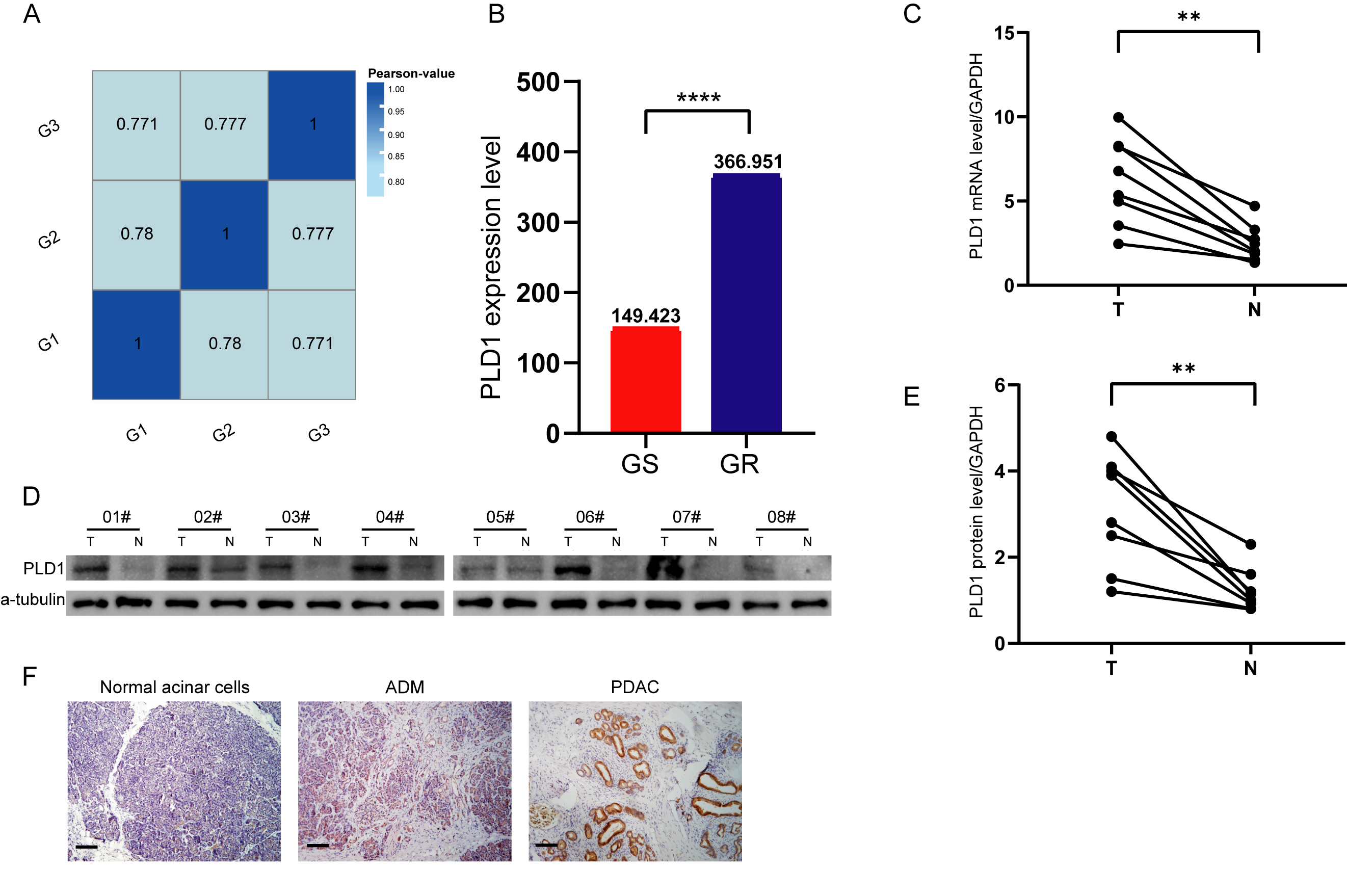

Supplement: Supplementary file 1 [file cbm-20-599-s001.zip › cbm-20-599-s004.tif]

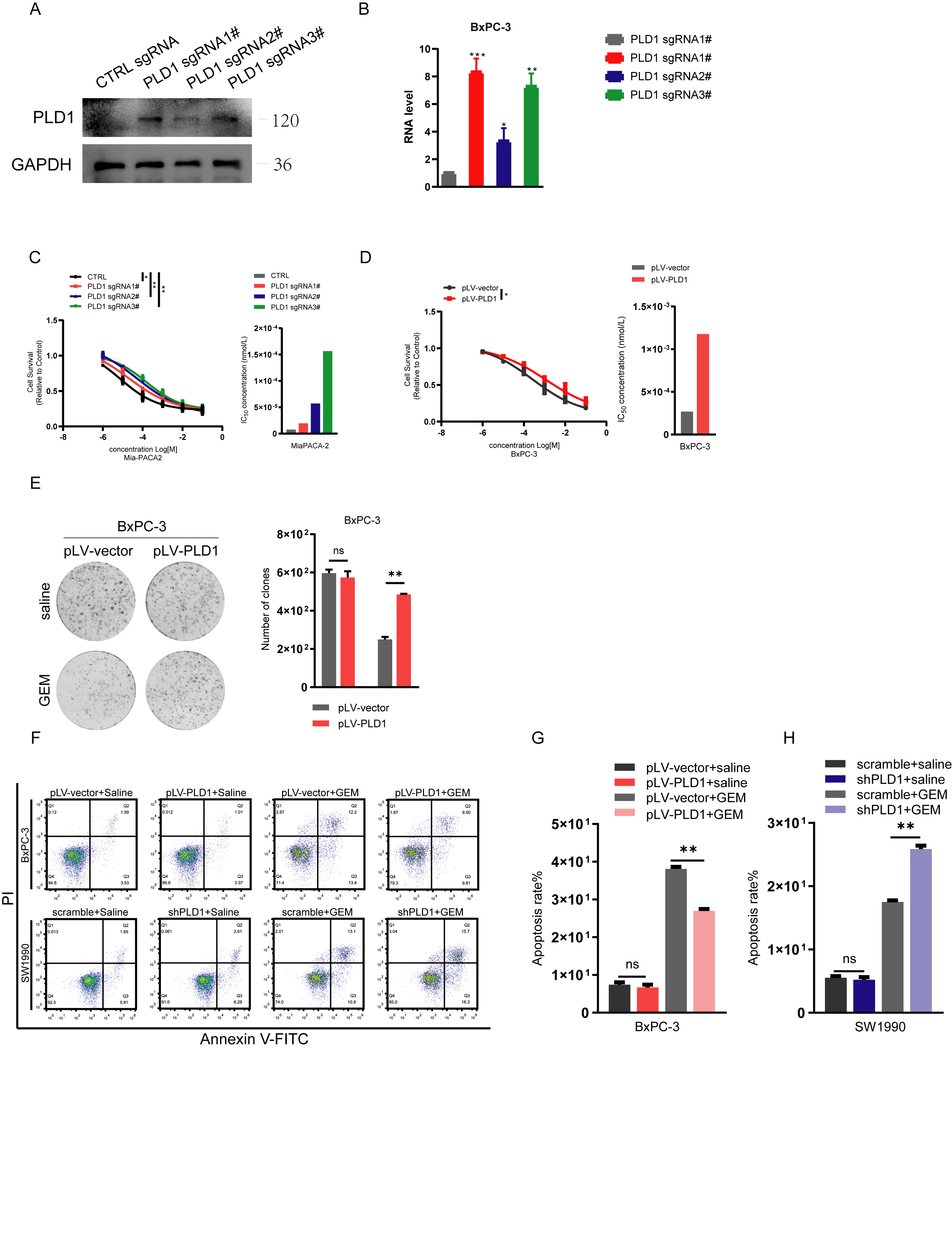

Supplement: Supplementary file 1 [file cbm-20-599-s001.zip › cbm-20-599-s005.tif]
